# Modeling behavior dynamics using computational psychometrics within virtual worlds

**DOI:** 10.3389/fpsyg.2015.01725

**Published:** 2015-11-06

**Authors:** Pietro Cipresso

**Affiliations:** Applied Technology for Neuro-Psychology Lab, IRCCS Istituto Auxologico ItalianoMilano, Italy

**Keywords:** computational psychometrics, virtual reality, behavior dynamics, modeling, psychometrics, simulation, computational intelligence

## Abstract

In case of fire in a building, how will people behave in the crowd? The behavior of each individual affects the behavior of others and, conversely, each one behaves considering the crowd as a whole and the individual others. In this article, I propose a three-step method to explore a brand new way to study behavior dynamics. The first step relies on the creation of specific situations with standard techniques (such as mental imagery, text, video, and audio) and an advanced technique [Virtual Reality (VR)] to manipulate experimental settings. The second step concerns the measurement of behavior in one, two, or many individuals focusing on parameters extractions to provide information about the behavior dynamics. Finally, the third step, which uses the parameters collected and measured in the previous two steps in order to simulate possible scenarios to forecast through computational models, understand, and explain behavior dynamics at the social level. An experimental study was also included to demonstrate the three-step method and a possible scenario.

## Introduction

In a recent article, Gomez-Marin et al. (2014) defined animal behavior as “the macroscopic expression of neural activity, implemented by muscular and glandular contractions acting on the body, and resulting in egocentric and allocentric changes in an organized temporal sequence” (p. 1456). This definition highlights the complexity of behavior in terms of “systemic emergence” from micro to macro elements (Serra and Zanarini, 2012; Liu et al., 2013; Reynolds, 2014).

Modeling behavior is possible at the micro level through computational neuroscience and at the macro level (society) through computational psychology (e.g., social network analysis and mathematical modeling). However, the real problem for researcher is to understand to what extent realistic behavior can be modeled, as behavior is relational, dynamic, and multidimensional (Gomez-Marin et al., 2014). These three attributes are essential in order to understand the complexity of modeling behavior.

Human behavior is relational in the sense that humans, interacting, act in a context, within a world. These interactions are not static but rather exist and continuously change in time and space. Furthermore, behavior is manifested in multiple forms, such as gestures, expressions, and psychophysiological changes.

Due to the complex nature of behavior (Bieri, 1955; Cambel, 1993; Robertson and Combs, 2014), its modeling cannot be based on a combination of variables in equations (Cushing, 2013; Puccia and Levins, 2013). Instead, the relational, dynamic, and multidimensional nature of behavior must be studied under the umbrella of complex systems, using computational science (Thelen and Smith, 1996, 2007; Vespignani, 2012; Goertzel, 2013; Liu et al., 2013). From this perspective, it is possible to further investigate the behavior components at the micro level that affect behavior at the macro level. Thanks to the significant steps taken in recent decades in computational science (Conte et al., 2012), we can theorize elegant models and rich simulations within *in silico* experiments (Batut et al., 2013). However, to bridge these models with the real world and real data remains difficult.

One of the biggest challenges in this area is related to the reliance on big data analytics, which makes it necessary to extract a small amount of information from a huge amount of collective behavior (Wu et al., 2014). However, this approach, even though its popularity is growing, cannot yet be considered efficient alone, due to the high level of complexity, and the lack of control of experimental conditions, that are essential to the perspective of a model calibration (Kitchin, 2014).

### Research questions and aim of the method

Computational simulation can be used to analyze the behavior dynamics at a macro level; however, the input we give to the models depends on how we define the behavior at a micro level. For example, if we want to study a swarm, then we can observe it as a whole, otherwise we can analyze how each single component of the storm interacts with the other. The idea of complexity science is actually very simple and relies on the observation and manipulation of micro behavior to understand what simple rules bring to a group dynamic. In the case of fire in a building, how does an individual interact with others, and above all, with the crowd? To answer to this question, we need to make some hypotheses about each individual's behavior, but the real problem is that the behavior, as stated, is relational, dynamic and multidimensional. So, practically, three main questions arise:

1. How do we study human behavior as it relates to specific situations that could also be impossible to replicate (such as the case of fire in a building) or experimentally manipulate?
2. How is it possible to measure the parameters that provide us information about the behavior dynamics of one, two, or many individuals?
3. How can we include all the possible parameters of a behavior dynamics in a model to be used to forecast collective behavior at the macro (social) level?

Answering each one of these research questions, we have a three-step method for modeling behavior dynamics. In the following I propose Virtual Reality (VR) as the elective method to study human behavior related to specific situation, opportunely emulated. Then I explain how to measure behavior parameters to study the dynamics of one, two, and many individuals. Finally I use computational models for simulation of dynamic behavior in a population. An experimental study is also included as a first demonstration of the three-step method.

### Simulation, emulation, and real behavior

**Figure 1:**
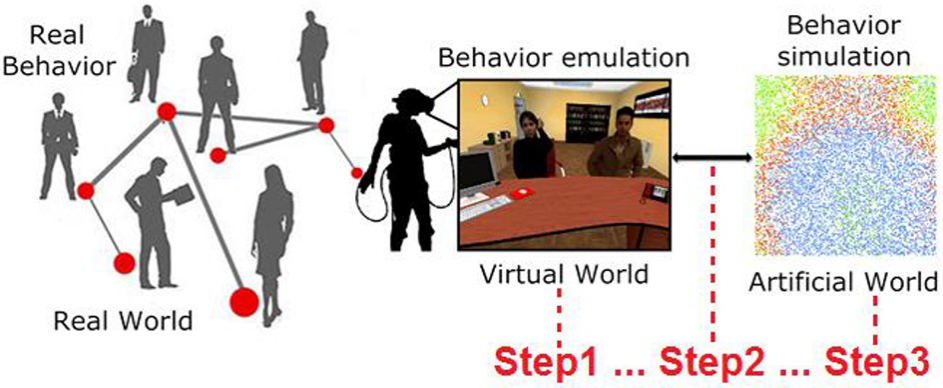
**Real behaviors can be emulated in VR by creating specific situations to elicit them (Step1)**. Once manifested, such behaviors can be measured and included (Step2) in a computational model to run an artificial simulation from the bottom up to study behavior dynamics (Step3).

In the current literature, there is still confusion over the definition of a simulation (Cacciabue, 2013; Robinson, 2014). In the computer science field, most researchers define VR as a simulation (Biocca and Levy, 2013; Earnshaw, 2014). In psychology, the situation is more complex, since mental imagery or the real generation of a situation with actors are considered simulations as well as VR (Moulton and Kosslyn, 2009). For these reasons, in this article, under the umbrella of complex systems (Bar-Yam, 2002) by using computational psychometrics (Cipresso et al., 2015), our aim was to create interconnections among real behaviors, by emulating them in VR, in order to simulate behaviors in an artificial world (Figure 1).

**Figure 2:**
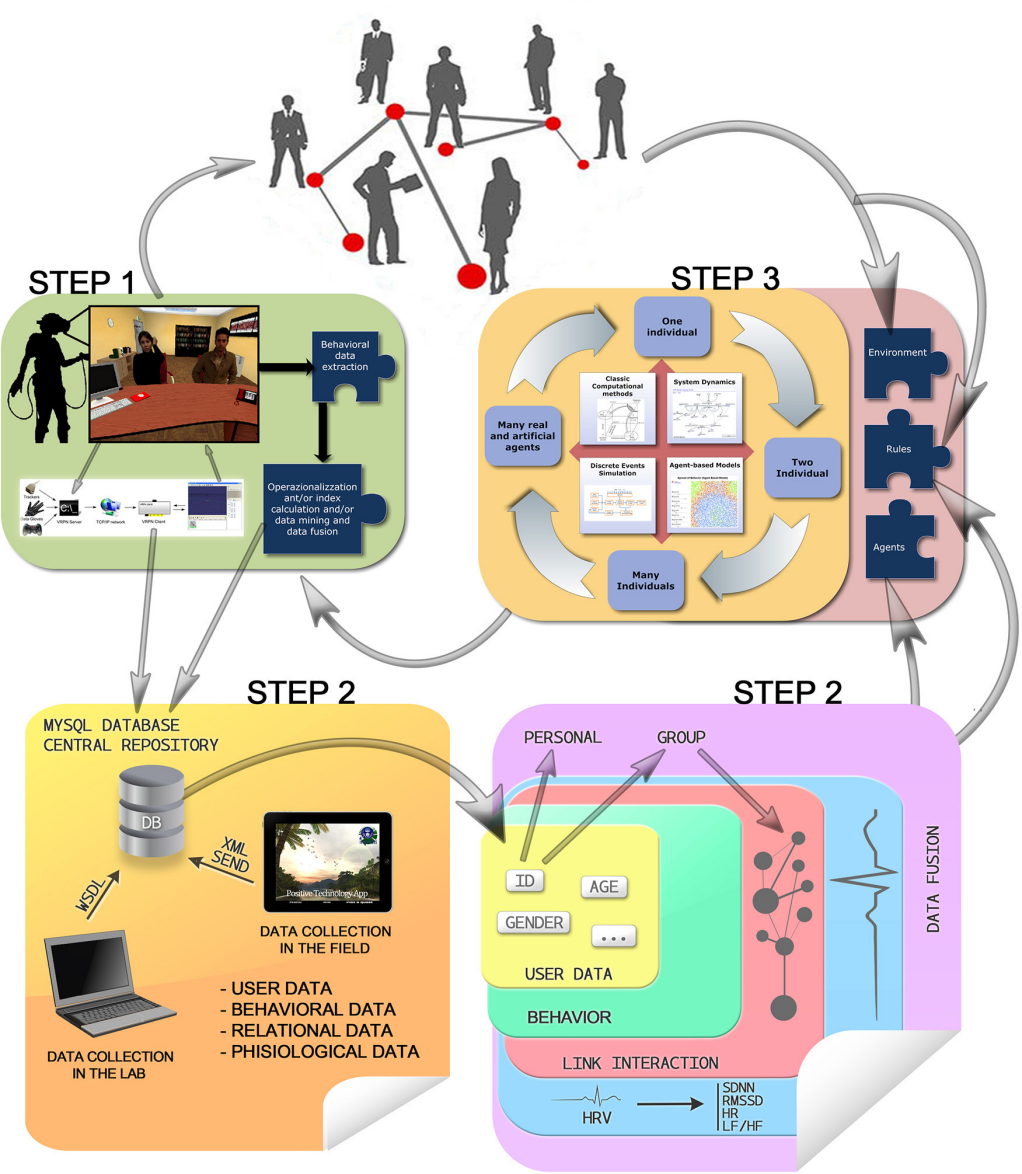
**Behavioral, relational, physiological, and user data are collected in lab settings (e.g., by means of VR) or in the field (e.g., by means of mobile applications), contributing to feed the model's main input (environment, rules, and agents) and feedbacking the users with virtual environment changes, which in turn contribute again to feed the models, up to the system homeostasis**.

The use of Virtual Reality (VR) platform is interesting also because it is possible to use measures micro level variables. In particular, spatial, and temporal variables (system log with route and timestamps), physiological variables (integration of biosensors with the VR platform, using a communication protocol, with signals recorded with logging of events, routes, and timestamps), and relational variables (using questionnaires integrated in the VR platform and logging events such as social connections). An extensive plan of the three-step method is represented in Figure 2.

The three-step method requires a formal measure of interactions and this can be a very difficult issue. To synthesize the measurement of interactions the following table (Table 1) provides possible approaches for its investigation.

**Table 1:** **Modeling behavior dynamics**.

**Number of individuals**	**Approaches**	**Instruments and theories**	
One individual $\left\{ \begin{array}{l} \to ■\;Input\;system\\ ■\to Output\;system \end{array}\right.$ *TCP/IP port in NeuroVirtual 3D to create a bridge between real behavior and virtual environments*	Psychology Psychophysiology Systems HCI Biofeedback Virtual Reality	• Presence • Virtual environments • Social and cultural cues	Environment affects my behavior
		• Behavior manifestation • Behavior influence on Virtual Environment	My behavior changes the environment
Two individuals ■⋯■*Virtual Reality as a tool*	Complex systems	$\begin{array}{l} \text{Entropy}\\ \text{Information}\\ \text{Characterization} \end{array}\}$ Mutual exchange of behavior (contagion)	
Many individuals *Networked and Ubiquitous*	Mathematical Theoretical Empirical Social Ubiquitous	Virtual reality and mobile app Random networks and strategic models Network structure System dynamics Discrete event processes	
		• Propagation of behaviors	
		• Diffusion processes	
Many individuals and artificial complex networks *NeuroVirtual 3D with simulations and Artificial Life*	Psychology Artificial Simulation based Artificial Intelligence Virtual Intelligence	$\begin{array}{l} \text{Agent-based models}\\ \text{Real-based Modeling} \end{array}\}$ Dynamic processes	

Approaches, instruments, and theories considered on the basis of the number of individual taken into account: from only one individual interacting with environment to many real and modeled individual.

## Human behavior related to specific situations

Classic methods to emulate specific situation relies on exposure and recall of experience though imagery, in particular by the means of text, video, and audio (Sadoski and Paivio, 2012; Paivio, 2013; Richardson, 2013; Reisberg, 2014). Unfortunately these methods have several problems: mainly they require a big ability in imagining the specific situation (Finke, 2014) and they are out of the full experimental control and so they are often passive exposure methods (Paivio, 2013). Moreover, the logging of specific events or activity and active interaction with the situation, is impossible.

To overcome these limitation, I propose the use of an advanced platform of VR, which is able on the one hand to elicit behavior in a replicable setting and on the other hand to extract precise parameters, while keeping the experimental conditions controlled. Using virtual environments, it is possible to create replicable and standardized settings within which real humans can experience situations eliciting the behavior that manifests in the subjects in relational, dynamic, and multidimensional frameworks. Moreover, it is possible to interconnect simulated with real behavioral data, calibrating the computational models by using real empirical data collected in a controlled setting.

Virtual environments are particularly suitable for behavioral studies; in fact, within these environments it is possible to satisfy all the experimental requirements linked to the relational, dynamic, and multidimensional attributes that are the core framework of behavior.

As behavior is relational, it is necessary to consider the “affordances” as the opportunity to behave within a context (Gibson, 1977; Schuemie et al., 2001; Dalgarno and Lee, 2010).

A key concept when contextualizing behavior within virtual environments is that of presence, or the sense of “being there” (Mantovani and Riva, 1999; Riva et al., 2003). If an individual immersed in a virtual environment feels like he/she is actually “there,” then he/she will behave in a realistic way, and his/her manifested behaviors will be the macroscopic expression of deeper intentions (Riva et al., 2015).

As presence is a prerequisite to manifest behavior in a virtual environment, it is necessary to understand how to assess presence. As Ijsselsteijn et al. (2000), Ijsselsteijn et al. (2001), and Biocca et al. (2003) underlined, there is not a single unified model to assess the sense of presence in a mediated environment, although different approaches have been suggested, such as subjective self-report measures (Slater and Garau, 2007), continuous presence assessment (Ijsselsteijn et al., 1997), analysis of postural and gestural responses (Freeman et al., 2000; Giakoumis et al., 2012), psychophysical methods (Stanney et al., 1998), physiological indexes (Wiederhold et al., 2000; Mikropoulos, 2001; Baumgartner et al., 2006; Schilbach et al., 2006), and many others, as suggested by Schultze (2010). Thus, as in the case of behavior, it is crucial to consider presence as relational, dynamic, and multidimensional. Practically, behavior manifests within presence as the expression of one's own intentions.

Using advanced VR platform, we can generate several prototypical situations to elicit behavior within a controlled setting, which will enable us to extract the main parameters that will allow us to simulate and study behavior in a more efficient way.

### Behavior dynamics within virtual environments

The huge growth in the availability and capabilities of current commercial computers has made significant development possible in the VR field. Currently, it is possible to purchase a complete VR system with a head-mounted display (HDM) for visualization and head tracking for < US $2000. Availability, in terms of technologies and costs, has allowed for a considerable diffusion of VR in different fields, from industrial application to cyber-therapy and clinical practice.

However, unfortunately, the costs to produce a virtual environment are still high, requiring teams of technicians and psychologists working closely to build each environment step-by-step, often for use in only a single one experiment. More data require a complex process to be extracted.

To overcome these limitations, we propose the use of NeuroVirtual 3D (www.neurovirtual.eu), an advanced platform that we developed for experimental and computational psychology (Cipresso et al., 2014b). The platform makes psychological settings easy to manipulate by creating a virtual environment through a simple process of “drag and drop” 3D objects, pictures, video clips, and other items. Using a simple wizard, researchers are able to define properties, tasks, and collisions based on proximity, mouse clicks, key presses, or other button functions, as well as with the use of a Kinect. Finally, the platform has an input/output communication port based on TCP/IP protocol, which allows for the creation of a bridge between the virtual environment and the real world. TCP/IP protocol can also be used to define communication between VR experiments and computational simulations, making the platform a bridge between real and modeled behavior (Cipresso et al., 2014b).

Within this premise, virtual environments need to be interactive, offering more than just a “box” in which to move without a scope. In an experimental perspective, NeuroVirtual 3D gives the opportunity to define “affordances” for experimental designs. It is possible to define stimulus presentation in a more “ecological” way, by using tridimensional virtual environments and objects that can be observed like in the real worlds, instead of 2D static images. It is also possible to create complex interactions where objects can have or lack certain physical properties, such as gravity. Also, the environment and its conditions might change on the basis of experimental conditions, and this makes VR more powerful than actual reality for behavioral science experiments. For example, in protocols about spatial abilities, the environments can continuously change, and the physical properties, such as the walls in a maze, which are constrained in a real environment, can be manipulated (moved or removed) in a virtual one (Cipresso et al., 2014a).

Another key aspect to consider is interaction with others, including simulated and/or real individuals within the virtual environment. In the first case, avatars, or video clips with prototypical situations can be used to elicit a certain behavior in the viewer. Real video in virtual environments can be highly realistic, because it can be set to start on proximity; that is, the video can begin when subjects are close to it, giving the impression that the video is sensible to and responding to the subjects' actions (for example, an apple can fall from a tree when one is close to it). Moreover, video clips can use a Chroma-key technique (creating an invisible background), so that within NeuroVirtual 3D they appear as real persons or real objects. In the second case, real others can be included as avatars and can have a specific task similar to a real laboratory or real-life setting.

This huge flexibility of virtual environments offers a significant opportunity for measuring and, above all, for modeling behavior. In fact, using such environments we can do things that are not possible in real settings, and we can extract simple or complex information connected to the observed behavior, such as response to standardized tasks, psychophysiological parameters (difficult to record in real settings), route in the virtual environment (spatial navigation), body motions (head, eye, hand, and body tracking), and several other measures that are easy to collect with the use of NeuroVirtual 3D (Cipresso et al., 2014a).

## Modeling behavior dynamics considering only one individual in virtual environments

An individual can be considered a Complex Adaptive System (CAS) (Bar-Yam, 1997). At the macro level, a CAS shows complexity and emergence properties, thanks to adaptivity, self-organization (attraction and repulsion processes), stigmergy, autocatalysis, syntropy, self-similarity, and so on. As the environment changes, a CAS has the capacity to adapt to it. Thus, when a perturbation occurs in the systems, a CAS reconfigures itself without substantial loss of its own functionality; of course, this depends on the resilience of the system (Miller and Page, 2007; Buckley, 2008).

A crucial property of CAS, particularly in individuals, is homeostasis (Buckley, 2008), which refers to the capacity of a system to regulate its own internal values to tend toward a stable equilibrium. To act this regulation we have to introduce the concept of feedback. Basically, feedback enters the system and modifies its dynamics. There are two different kinds of feedback:

- Positive feedback, with which the changes are amplified, causing the system to be unstable; and
- Negative feedback, with which the changes leads the system toward homeostasis.

In the CAS field, it is common to speak about “the edge of the chaos.” This idea was originally developed by Packard and Langton through computational experiments. The idea is that simple systems are static, while too-active system are chaotic; thus, complexity lies “on the edge” between these two extremes, where the systems have the capacity for emergent computation (Miller and Page, 2007).

Already in the early 1950s, Friedrich Von Hayek, Nobel Prize recipient in economics, had introduced the idea of “spontaneous order.” This spontaneous emergence of order out of seeming chaos and other thoughts related to CAS resulted in a vivid evolution of mathematical and computational techniques and models (Miller and Page, 2007; Buckley, 2008).

Considering only one individual in a virtual environment, two main aspects can be taken into account: presence, which refers to our sense of being into a virtual environment, and interaction with the environment, which also includes the objects within it.

**Figure 3:**
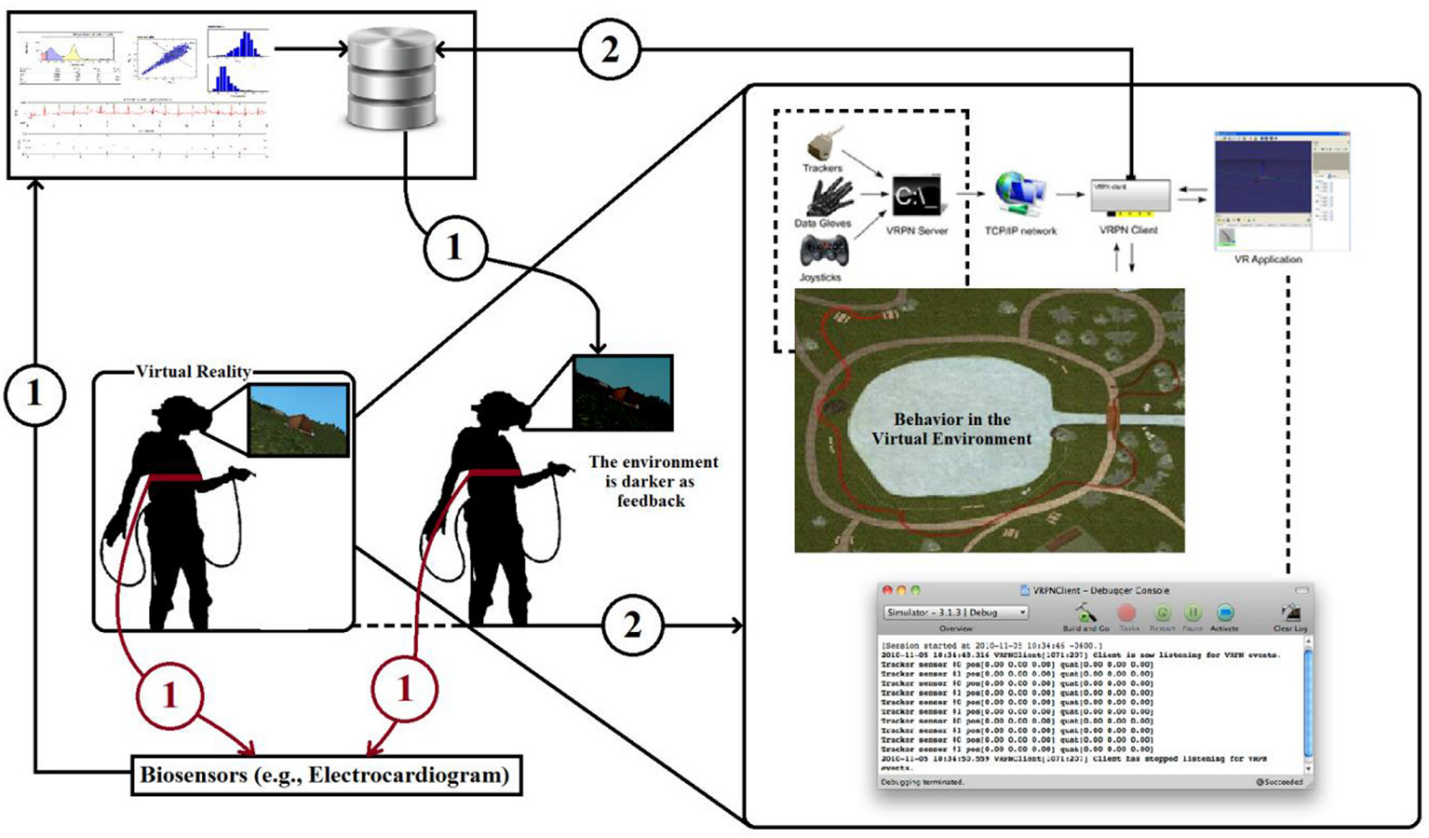
**Loop (1) represents the classic biofeedback where the environmental light changes continuously on the basis of physiological parameters**. Loop (2) represents an extended version in which the individual's behavior contributes to the changes in the virtual environment. Loop (2) may also include information from other individuals (group biofeedback). Eventually, these individuals may have relationships (networked biofeedback).

NeuroVirtual 3D allows for a full interaction of the users in the virtual environment and is able to interconnect with the external (real) world by including elements of it in the VR setting. Thanks to a VRPN (Virtual-Reality Peripheral Network) protocol, it is possible to integrate with NeuroVirtual 3D many devices, including human behavior, once coded. One of the most useful integrations is the implementation of a “biofeedback” connector, which allows users to change the environment or specific objects with psychophysiological indexes measured through the use of specific biosensors. In particular, heart rate variability indexes can be used to monitor emotional behavior in specific situations. In Figure 3, it is possible to see the loop (1) where an individual immersed in a virtual environment is connected with an electrocardiogram (ECG) taken through a strip sensor on the chest of the user. Using this technique, users are able to see the light in the environment (from darker to lighter and vice versa) representing their internal behavior. Biofeedback has already come to be seen as a gold standard in clinical use (Green and Green, 1977; Yates, 1980; Schwartz and Andrasik, 2003), with several applications, prior to its use within virtual environments. The challenge in VR is to improve the ecological validity of the treatment and thus to be more effective in the cure (Green and Green, 1977). Group biofeedback and networked biofeedback in Figure 2 are already possible with NeuroVirtual 3D but still have yet to be tested in a randomized controlled trial. The integration of NeuroVirtual 3D with the real world also provides an excellent instrument for behavioral science. The challenge is to understand behaviors and include them in the virtual environments in order to provide direct feedback for the self and others. A closed loop between the individual behavior changes and the virtual environment modification is one of the most important avenues to explore for complex system modeling and the interaction between the calibrated models and real human behavior.

## Modeling behavior dynamics between two individuals

As we explained before, behavior is relational; thus, it is natural to inquire about the mechanics of the relationship of behavioral changes between two individuals in a mutual interaction. Since individuals are complex systems with non-linear behavior (i.e., non-linear reactions to others' actions), it is necessary to model behavior under the umbrella of complex science, defining the reciprocal behavior through information theory models. One of the first contributions in this sense has been described by Shannon and Weaver in their model of communication, further explained in Cover and Thomas (Shannon and Weaver, 1959; Cover and Thomas, 2012). The same model can be used to derive the behavior mechanics in formal terms.

We can define a measure of the mutual exchange of behavior (contagion) between two individuals in term of a probability *p*. We will have a measure defined *I(p)* with many properties: *I(p)* ≥ 0, i.e., contagion is non-negative; *I(p*_1_*p_2_*)* = *I(p*_1_*)* + *I(p*_2_*)*, i.e., if the join probability of two independent events occur, then the contagion is the sum of these two information; *I(p)*, the contagion, need to be a monotonic and continuous function of the probability *p*; *I(1) = 0*, i.e., for an event with probability *1* (a sure event) we obtain no one behavior. Therefore, *I* (*p*2) = *I* (p*p) = *I* (*p*)+*I* (*p*) = 2*I (*p*), by induction, (*p*^*n*^) = n*I (*p*), $I\left(p\right)=\;I\left({\left(p^{\frac{1}{m}}\right)}^{m}\right)\;=\;m\;*\;I\left(p^{\frac{1}{m}}\right)$, thus, $I\left(p^{\frac{1}{m}}\right)=\frac{1}{m}*I\left(P\right),$ in general terms, $I\left(p^{\frac{n}{m}}\right)=\;\frac{n}{m}*I\left(p\right)$ and, thanks to continuity, *I* (*p*^*a*^) = a*I (*p*) for 0 < *p* ≤ 1 and *a*>0, *a*∈ℝ. Thus, we obtain, $I\left(p\right)=\;-{log}_{h}\left(p\right)=\;{log}_{h}\left(\frac{1}{p}\right)$ for a constant *h*>0. This base (*h*) define the unit that we are using. For different *h*, in the base of logarithm of contagion measure *I(p)*, we will get: *log*_2_ units are bits, derived from binary; *log*_3_ units are trits, derived from trinary; *log*_*e*_(= *ln*) units are nats, derived from natural logarithm; *log*_10_ units are Hartleys, derived from a researcher in this field. Roughly speaking the amount of information necessary to describe a system, define the complexity of this.

Suppose to have a sequence {*s*_1_, *s*_2_, …, *s*_*n*_} provided independently by a source, with probabilities {*p*_1_, *p*_2_, …, *p*_*n*_} respectively. We are interested in the weighted average quantity of contagion, obtained from each *s*_*i*_, for *i* = 1, …, *n*. Considering a long run (assume, N) we will have *N* observation, of course independent, and a total contagion I given by $I=\;\overset{n}{\underset{i=1}{\sum }}\left(N*p_{i}\right)*log\left(\frac{1}{p_{i}}\right)$; i.e., for each *s*_*i*_ (*i* = 1, …, *n*), $\frac{I}{N}=\left(\frac{I}{N}\right)\overset{n}{\underset{i=1}{\sum }}\left(N*p_{i}\right)*log\left(\frac{1}{p_{i}}\right)=\overset{n}{\underset{i=1}{\sum }}p_{i}*log\left(\frac{1}{p_{i}}\right)$. Since $\lim_{n\to 0}n*log\left(\frac{1}{n}\right)=0$, we will define $p_{i}*log\left(\frac{1}{p_{i}}\right)=0$, if *p*_*i*_ = 0.

We defined the contagion as function of probabilities of events *{p*_1_*, p*_2_*, …, p*_*n*_*}*. Say this set *P* and define the entropy of this distribution as $S\left(P\right)=\overset{n}{\underset{i=1}{\sum }}p_{i}*log\left(\frac{1}{p_{i}}\right)$. Generalizing S(P) in the continuous rather than in the discrete, we will obtain $S\left(P\right)=\int P\left(x\right)*log\left(\frac{1}{P\left(x\right)}\right)dx.$

Now we try to think to entropy in terms of “expected value.” Speaking of probability, considering the discrete distribution *{p*_1_*, p*_2_*, …, p*_*n*_*}*, we have *p*_*i*_≥0 and $\overset{n}{\underset{i=1}{\sum }}p_{i}=1$, where considering a continuous distribution *P(x)* we have *P* (*x*)≥0 and ∫*P* (*x*)*dx* = 1. Thus, defining the set *{h*_1_*, h*_2_*, …, h*_*n*_*} as H*, the expected value of H will be $E\left[H\right]=\overset{n}{\underset{i=1}{\sum }}h_{i}*p_{i}$ for discrete distribution *{p*_1_*, p*_2_*, …, p*_*n*_*}*, or *E*[*H* (*x*)] = ∫*H* (*x*)*P* (*x*)*dx* for continuous distribution *P(x)*. Considering previous results on entropy and contagion, we obtain *S* (*P*) = *E*[*I* (*p*)], i.e., the expected value of the contagion of our distribution is the entropy of the probability distribution.

A Shannon's basic model is composed of three parts: a source (who send a message), a channel (where the message run), and a receiver (who receive the message) (Shannon, 1948). In a more general model Shannon considers also the noise in the channel and decoding and encoding systems (Shannon, 1949). Shannon, in the first part of his paper, define a discrete model, assuming a sequence of *{h*_1_*, h*_2_*, …, h*_*n*_*}* letters, sent through a channel (disturbed by a noise) and then encoded, by the source. Then the receiver decode the symbols in information. Behavior follow the same modeling method where information are encoded and modeled as a sequences.

Assuming that the elements through the channel are single elements (like gestures), we could define an “alphabet” of this channel. In general we can consider a set of symbols *{r*_1_*, r*_2_*, …, r*_*n*_*}*, called *R*; considered this alphabet *A*, the encoder is a function *e*:*A*→*R*^+^, where *R*^+^ represent all the finite strings that we can obtain from *R.*

Let consider now the McMillan Kraft inequality $K=\sum_{i\;=\;1}^{n}\frac{1}{\beta^{l_{i}}}\leq 1\Leftrightarrow \exists \;!\;u\;:\exists \;ℒ\left(u\right)\in \left\{l1,\;l2,\;\ldots ,\;ln\right\},\;$i.e., we have only one code with length *{l*_1_*, l*_2_*, …, l*_*n*_*}* iif *K* ≤ 1 where *l*_*i*_ = | *e* (*a*_*i*_) |, *i* = 1, 2, …, *n*; *and a*_*i*_ ϵ* A*.

Let consider, now $k^{n}={\left[\overset{n}{\underset{i=1}{\sum }}\frac{1}{\beta^{l_{i}}}\right]}^{n}=\overset{nl}{\underset{i=1}{\sum }}\frac{N_{k}}{\beta^{k}},$ with *l = Max {l*_1_*, l*_2_*, …, l*_*n*_*}.* Of course $N_{k}\leq \beta^{k}$, thus $K^{n}\leq \overset{nl}{\underset{k=n}{\sum }}\frac{\beta^{k}}{\beta^{k}}=nl-n+1\leq nl.\;$Thus, *K* ≤ 1. Now define, for $0<G_{i}\leq 1,\;and\;\overset{n}{\underset{i=1}{\sum }}G_{i}=1$, $G_{i}=\frac{\beta^{-l_{i}}}{K}.$ Applying the Gibbs inequality, given *p*_*i*_ the probability to observe *h*_*i*_, we will obtain: $\overset{n}{\underset{i=1}{\sum }}p_{i}log\left(\frac{G_{i}}{p_{i}}\right)\leq 0$ or $\overset{n}{\underset{i=1}{\sum }}p_{i}log\left(\frac{1}{p_{i}}\right)\leq \overset{n}{\underset{i=1}{\sum }}p_{i}log\left(\frac{1}{G_{i}}\right).$

Note that $\text{S}\left(\text{B}\right)=\overset{n}{\underset{i=1}{\sum }}p_{i}log\left(\frac{1}{p_{i}}\right)$ represent the source's entropy, thus $S\left(B\right)\leq \overset{n}{\underset{i=1}{\sum }}p_{i}\left[log\left(K\right)-log\left(\beta^{-l_{i}}\right)\;\right]=log\left(K\right)+\overset{n}{\underset{i=1}{\sum }}p_{i}l_{i}log\left(\beta \right)\leq log\left(\beta \right)\overset{n}{\underset{i=1}{\sum }}p_{i}l_{i}.$ Thus, *S* (*B*) ≤ *Llog*(β), where $L=\overset{n}{\underset{i=1}{\sum }}p_{i}l_{i}$. So the entropy provide a lower-bound on the efficiency of the encode system. We need to shape a better model of probability to improve this schema. Let us consider *F = {f*_1_*, f*_2_*, …, f*_*n*_*}*, containing input symbols and *G = {g*_1_*, g*_2_*, …, g*_*m*_*}*, containing output symbols, where can also be *m*≠*n*. Since we don't know which symbol *f*_*i*_ generated the symbol *g*_*j*_, the characterization of the channel is given by the set {*P* (*f*_*i*_ | *g*_*j*_)}.

Now we can define the mutual contagion $I\left(f_{i};g_{j}\right)=log\left(\frac{1}{P\left(f_{i}\right)}\right)-log\left(\frac{1}{P\left(f_{i}|g_{j}\right)}\right)=log\left(\frac{P\left(f_{i}|g_{j}\right)}{P\left(f_{i}\right)}\right),$ where *P* (*f*_*i*_) is an “a priori” estimate.

Thus: *I* (*f*_*i*_; *g*_*j*_) = *I* (*g*_*j*_; *f*_*i*_); *I* (*f*_*i*_; *g*_*j*_) = *log* (*P* (*f*_*i*_ | *g*_*j*_))+*I* (*f*_*i*_); *I* (*f*_*i*_; *g*_*j*_) ≤ *I* (*f*_*i*_); *P* (*f*_*i*_; *g*_*j*_) = *P* (*f*_*i*_)*P (*g*_*j*_)⇒*I* (*f*_*i*_; *g*_*j*_) = 0.

Now since $I\left(F;g_{j}\right)=\underset{i}{\sum }P\left(f_{i}|g_{j}\right)*I\left(f_{i};g_{j}\right)=\underset{i}{\sum }P\left(f_{i}|g_{j}\right)*log\left(\frac{P\left(f_{i}|g_{j}\right)}{P\left(f_{i}\right)}\right),\;$ and $I\left(f_{i};G\right)=\underset{i}{\sum }P\left(f_{i}|g_{j}\right)*log\left(\frac{P\left(g_{j}|f_{i}\right)}{P\left(g_{j}\right)}\right),\;$we will have $I\left(F;G\right)=\underset{i}{\sum }P\left(f_{i}\right)*I\left(f_{i};G\right)=\;\underset{i}{\sum }\underset{j}{\sum }P\left(f_{i};g_{j}\right)*log\left(\frac{P\left(f_{i}|g_{j}\right)}{P\left(f_{i}\right)P\left(g_{j}\right)}\right)$ = *I* (*G*; *F*).

Then, *I* (*F*; *G*)≥0, *I* (*F*; *G*) = 0⇔*P* (*F, G*) = *P* (*F*)*P (*G*), and I is symmetric in *F* and *G* (*I* (*F*; *G*) = *I* (*G*; *F*)).

Furthermore, $S\left(F\right)=\overset{n}{\underset{i=1}{\sum }}P\left(f_{i}\right)*log\left(\frac{1}{P\left(f_{i}\right)}\right),S\left(G\right)=\overset{n}{\underset{i=1}{\sum }}P\left(g_{j}\right)*log\left(\frac{1}{P\left(g_{j}\right)}\right),S\left(F|G\right)=\overset{n}{\underset{i=1}{\sum }}\overset{m}{\underset{j=1}{\sum }}P\left(f_{i}|g_{j}\right)*log\left(\frac{1}{P\left(f_{i}|g_{j}\right)}\right)$, $S\left(F,G\right)=\overset{n}{\underset{i=1}{\sum }}\overset{m}{\underset{j=1}{\sum }}P\left(f_{i},g_{j}\right)*log\left(\frac{1}{P\left(f_{i},g_{j}\right)}\right)$, *S* (*F, G*) = *S* (*F*)+*S* (*G* | *F*) = *S* (*G*)+*S* (*F* | *G*), *I* (*F, G*) = *I* (*F*)+*I* (*G*)−*I* (*F, G*) = *I* (*F*)−*I* (*F* | *G*) = *I* (*G*)−*I* (*G* | *F*)≥0, thus, we can write the mutual contagion *I* (*F, G*) as a difference between the marginal entropy and the conditional entropy.

Thus, given the knowledge of *G*, the decreased uncertainty of *F* represents the mutual contagion.

For this reason we call *I* (*F, G*) mutual contagion. Now we can define the “channel capacity” as $C^{Max}={Max}_{P\left(f\right)}I\left(F;G\right)$. Now we will use a standard maximum entropy principle (MEP).

Let us call *s*_*r*_ (*r* = 1, 2, …, *K*) some characteristics at macroscopic-level. Let us assume that these are associated to characteristics at microscopic-level by $\underset{i}{\sum }f_{i}*s_{i}^{\left(r\right)}=s_{r},$ onstrained by *f*_*i*_≥0 and $\underset{i}{\sum }f_{i}=1$: subjected to this constraints we have to maximize $\underset{i}{\sum }f_{i}*log\left(\frac{1}{f_{i}}\right)$, the entropy.

Thus, a general solution, is $f_{i}=exp\left(-\lambda -\underset{r}{\sum }\lambda_{r}*s_{i}^{\left(r\right)}\right),\;$where λ_*r*_ represents the standard multiplayer of Lagrange.

We can define $H\left(\lambda_{1},\lambda_{2},\;\ldots ,\lambda_{K}\right)=\underset{i}{\sum }exp\left(-\underset{r}{\sum }\lambda_{r}*s_{i}^{\left(r\right)}\right)$.

Thus, *e*^λ^ = *H* or λ = *ln* (*H*).

The mathematical analytics allows researcher to study the dynamics in a formal and elegant way, but unfortunately a model calibration to real behavior is very complex, being more adapted to a theoretical approach to computational communication than to real behavioral systems. However, the above equations represent the fundamental basis for the statistical mechanics approach, which we analyze below.

## Modeling behavior dynamics among many individuals

### Modeling behavior dynamics through “difference equation”

There are many ways to model behavior dynamics in complex systems (Bar-Yam, 1997). However, it is important to keep in mind that a model is only a representation of the real world; thus, we must first question the usefulness of such models. In conjunction with this more general question, we must consider the rationale we presented for CAS. In fact, we are interested in analyzing the emergence of general properties that could arise from possible network structures or, more generally, the properties that could arise from the interaction of simple rules (e.g., rules about behavior transmission). The idea is to start from simple models and then theorize about the behaviors that may occur in complex systems and networks.

The initial model of behavior dynamics is described by a difference equation. This model is similar to the classic Bass diffusion model (Bass, 1969, 2004; Jackson, 2008), an early model that has been very influential and is still used today.

We can begin by considering the first parameter to capture the response to environmental stimuli (e.g., political situations, general events, and media), spontaneous contraction (self-induced behavior), and other parameters that capture social and peer effects (behavior contracted by social interaction and imitation). In mathematical terms, considering discrete time *t*, and *S* (*t*) as the fraction of individuals who contracted behavior by time *t*, we have: *S* (*t*) = *S* (*t*−1)+α (1−*S* (*t*−1))+β (1−*S* (*t*−1)) *S* (*t*−1). In this difference equation, α is the rate of behavior contraction by spontaneous or environmental factors, while β is the rate of behavior contraction by social and imitation aspects. The term α (1−*S* (*t*−1)) is the rate of individuals who have not yet contracted behavior; β (1−*S* (*t*−1)) *S* (*t*−1) captures social and imitation processes, constituted by 1−*S* (*t*−1), which, again, is the fraction of individuals without behavior, and another expression, *S* (*t*−1), which is the fraction of individuals who contracted behavior and can therefore affect others or can be imitated. A continuous time version can be expressed as follows: $\frac{d\;S\left(t\right)}{dt}=\left(\alpha +\beta S\left(t\right)\right)\left(1-S\left(t\right)\right).$ Considering α > 0 and *S* (0) = 0, we have $S\left(t\right)=\frac{1-e^{-\left(\alpha +\beta \right)t}}{1+\frac{\beta }{\alpha }e^{-\left(\alpha +\beta \right)t}}.$ Now, let us try to determine the shape of the curve dynamics. At the first step, there are no behavior dynamics in individuals, and, consequently, there are no individuals to imitate. Thus, most of those who are the first to be affected will manifest behavior spontaneously (self-manifested behavior). From a mathematical point of view, when *S* (*t*) is approximately 0, the difference equation is approximated by α, the rate of behavior contraction by spontaneous or environmental factors. As time progresses, there are more individuals from whom behavior can be affected or imitated, and this leads to a higher spread rate. Then, at a certain point, there are many individuals from whom behavior can be affected or imitated, but there are not many people available to contract or imitate the behavior. Close to the last step (*t* = 1), there are no individuals who can be affected by others' behavior.

### Modeling behavior dynamics through system dynamics (SD) models

The difference equation approach is certainly interesting, but more often to model complex systems it is necessary to use more complex structures in order to define the feedbacks and different dynamics (slower or faster in terms of time) of different parts of a system. This is possible through a System Dynamics (SD) approach, which, by means of a computational approach, enables internal feedback loops and time delays that are able to change the entire system in a nonlinear way, as is required in complex systems (Karnopp et al., 1990).

**Figure 4:**
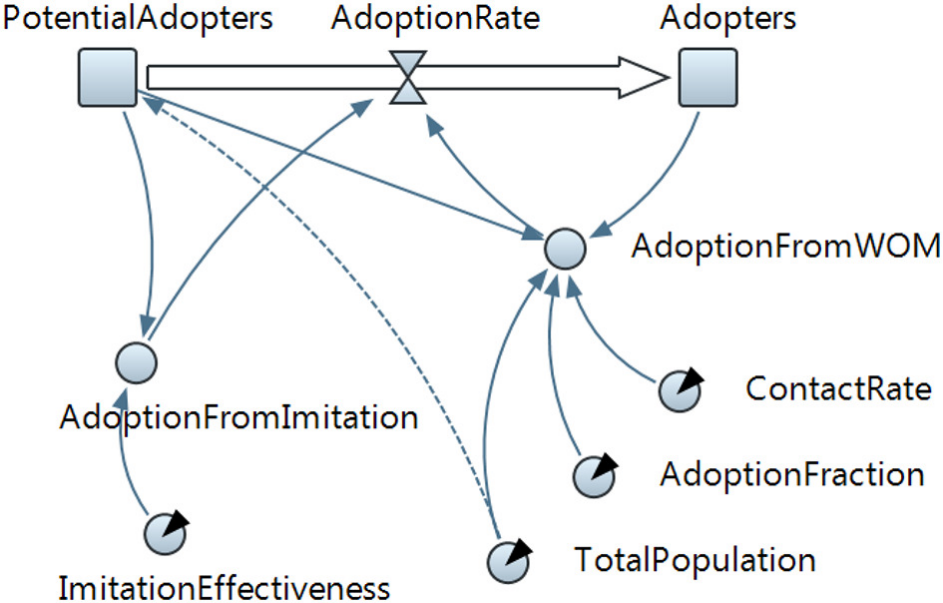
**The system dynamics presentation of the model is shown in this figure**. State variables are denoted with squares, flow with a “valve,” and dynamic variables with circles. Arrows denote causal dependencies in the model, with the dashed arrow indicating the initial condition.

The SD model in Figure 4 describes a behavior diffusion process. Potential adopters of a behavior are influenced to adopt the behavior by imitation and by word of mouth (WOM) from adopters (i.e., those who have already adopted the new behavior). Adoption of a new behavior driven by WOM is similar to an epidemic (AdoptionFromWOM is a dynamic variable in the diagram). Potential adopters come into contact with adopters (both are state variables and are represented with a square in the diagram) through social interactions. A fraction of these contacts result in adoption of the new behavior. Imitation thus causes a constant fraction of the potential adopter population to adopt each time period (AdoptionFromImitation is another dynamic variable in the diagram). Total Population, Contact Rate, Adoption Fraction, and Imitation Effectiveness are the fixed factors in the model.

The model is further explained in Figure 4 and Table 2. We need to analyze the model to decide how it can be described in terms of SD. We should distinguish the key variables of the model and their patterns of influence and then create “stock” and “flow” diagram of the model (Karnopp et al., 1990). When constructing a stock and flow diagram, we should consider what variables should be modeled with stocks, flows, or dynamic variables.

**Table 2:** **Model variables**.

**Fixed factors in the model:** • TotalPopulation • AdoptionFraction • ContactRate • ImitationEffectiveness
**Variables in the model:** • PotentialAdopters (initial value) = TotalPopulation • AdoptionFromImitation = PotentialAdopters * ImitationEffectiveness • AdoptionFromWOM = Adopters * PotentialAdopters * ContactRate * AdoptionFraction/TotalPopulation • AdoptionRate = AdoptionFromImitation + AdoptionFromWOM • d(PotentialAdopters)/dt = –AdoptionRate

Variables in the system dynamics model for behavior dynamics.

**Figure 5:**
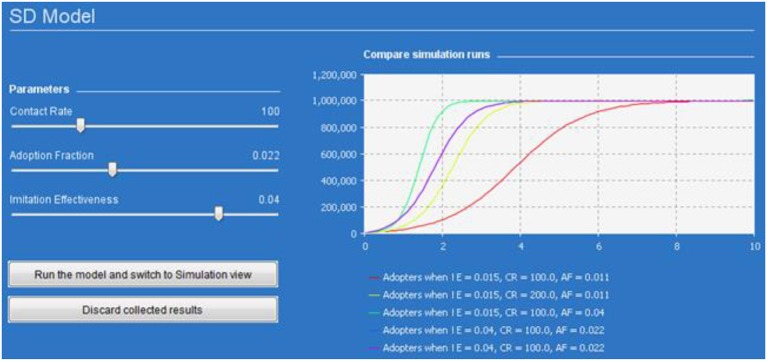
**Dynamics of one million adopters (vertical axis) in different simulation runs over time (horizontal axis)**. The colored lines corresponds to different parameters indicated in the legend.

**Figure 6:**
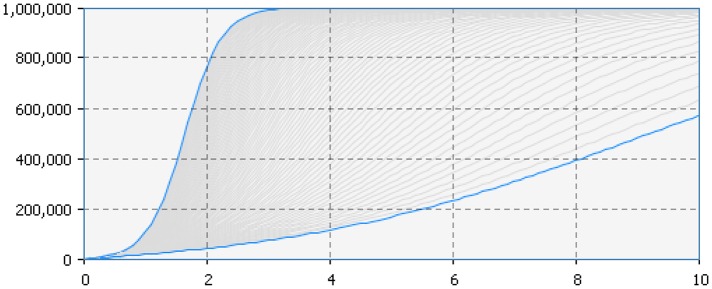
**Adopter (vertical axis) by time (horizontal axis)**. The figure represents all the range of possible dynamics from the faster (blue line in the top left hand side) to the slower (blue line in the bottom right hand side).

In particular, Figure 5 presents the SD simulation as executed in Java (see Supplementary Material for more information). Figure 6 presents all the range of possible dynamics from the faster to the slower.

Total population is defined for the simulation (in the present simulation, one million is the number of agents used), and contact rate can be estimated as an objective parameter (number of people with whom each individual is connected—generally between 100 and 300). However, “Adoption Fraction” and “Imitation Effectiveness” depend on the specific behavior to be modeled. For example, to set these two parameters for a specific behavior, we need to estimate the fraction of people in our population who manifest that behavior, which in turn depends on the individual propensity to acquire that behavior through contact with others. Moreover, we have also to quantify behavior adoptions arising from imitation, which in turn depends on the individual propensity to imitation. Estimating these two parameters (propensities to adopt and to imitate a behavior), we then have all the elements needed to run this model.

Individual propensity to adopt a behavior and individual propensity to imitate others' behavior can be estimated either in the lab or in the field. To estimate these parameters in the lab we can use multimedia files and experimental conditions and stimuli; however, to obtain a higher level of ecological validity of the behavior manifestation, we can use VR. The possible immersion in a realistic situation improves the experience and the psychometric properties in measuring the propensity to adopt and imitate a behavior. A platform like NeuroVirtual 3D allows us to integrate measures (behavioral, physiological, self-reported, etc.) and calculate an effective estimation of the needed propensities. It is also possible to create networked experiments with many users connecting to the platform, which allows for greater effectiveness in measuring the needed parameters. However, in a model, the best rule is to measure each parameter *ceteris paribus* so that their interaction is dealt with in the model dynamics without any other interference. Thus, only two experiments are necessary: one to estimate the propensity to adopt a behavior, in which a virtual situation is created for only this purpose, and one to estimate the propensity to imitate a behavior, with a virtual situation created to evaluate only the imitation of the behavior. Once both parameters have been estimated, we can run the model and start its calibration to estimate behavior dynamics in a population. Of course, this is a simplified model, which must be validated empirically with real data in order to be improved and calibrated furtherly.

Another interesting possible use of the model is that of comparing possible scenarios. Practically, if I want to test the behavior dynamics in two different conditions or within two different populations, I can estimate specific parameters for those cases, which in turn constitute a possible scenario of behavior dynamics. Different populations or different initial conditions can be compered in order to make better decisions.

### Modeling the behavior dynamics through SIR model with system dynamics

**Figure 7:**
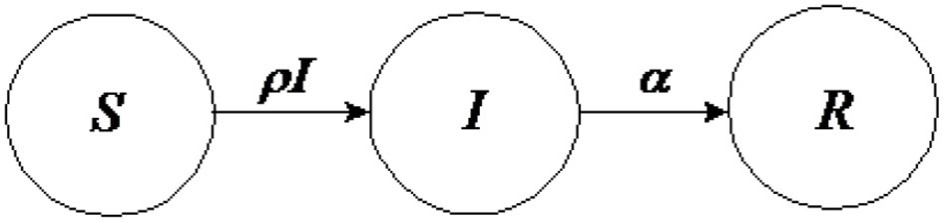
**Susceptible, Infected, Recovered (SIR) Model compartment diagram**.

Another model of behavior dynamics can be defined by using Bailey's (1975) “susceptible, infected, susceptible” (SIS) model. The idea of this model is to consider two states for a node, either “infected” or “not infected” (and thus susceptible to becoming infected). For the spread of behavior, it might be reasonable to use this model since an individual could recover from a behavior even though he or she is susceptible to engaging in that behavior again and again. Moreover, in general for many kind of behaviors, another characteristic that must be taken into account is that of spontaneous contraction of the behavior (i.e., self-induced behavior), which, in general, relates to a person's emotions (Hill et al., 2010). On the other hand, for many kinds of behaviors, it can be more interesting to use a “susceptible, infected, recovered” (SIR) model (Kermack and McKendrick, 1927), which can be represented by the following differential equations involving the variables S, I, and R (with S + I + R = 1) and their rates of change with respect to time t:

$$\begin{array}{l} \frac{dS}{dt}=-\rho SI,\;\frac{dI}{dt}=\rho SI-\alpha I,\;\frac{dR}{dt}=\alpha I, \end{array}$$

and the relative following “compartment diagram” (Figure 7).

**Figure 8:**
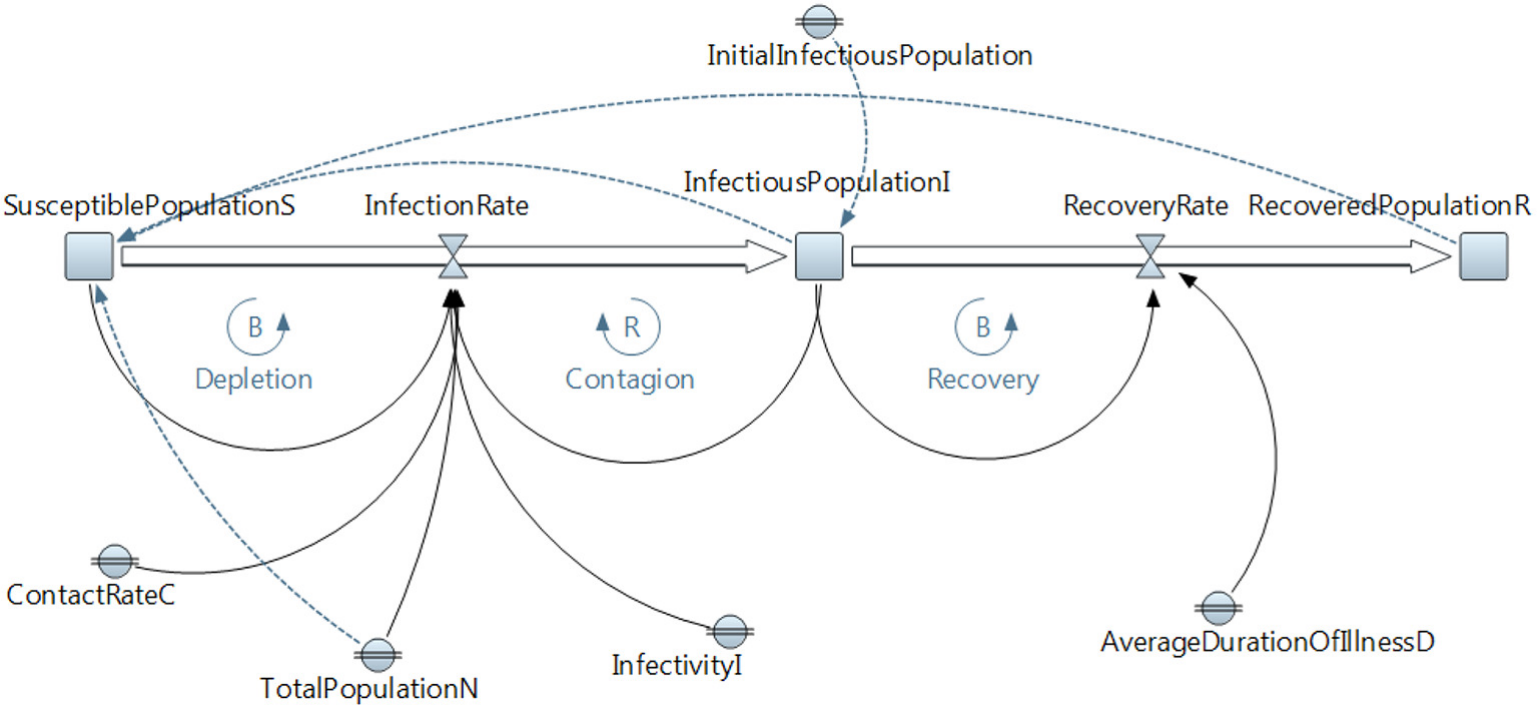
**Susceptible, Infected, Recovered (SIR Model) using system dynamics (SD)**.

SIR models have been widely used; however, a deeper modeling behavior is possible using SD. The SD model in Figure 8 and Table 3 describes behavior dynamics modeled using an SIR model with SD.

**Table 3:** **SIR Model variables**.

**Initial values of state variables:**
• SusceptiblePopulationS = TotalPopulationN − InfectiousPopulationI − RecoveredPopulationR
• InfectiousPopulationI = InitialInfectiousPopulation
• RecoveredPopulationR = 0
**Changes in state variables:**
• d(SusceptiblePopulationS)/dt = − InfectionRate
• d(InfectiousPopulationI)/dt = InfectionRate − RecoveryRate
• d(RecoveredPopulationR) = RecoveryRate
**Flows:**
• InfectionRate = ContactRateC * InfectivityI * SusceptiblePopulationS * InfectiousPopulationI/TotalPopulationN
• RecoveryRate = InfectiousPopulationI/AverageDurationOfIllnessD

Variables in the SD-SIR model for behavior dynamics.

## Modeling behavior dynamics in artificial complex networks

Even if we incorporate social and network aspects, by definition, the previous models do not explicitly take into account the network structure, which is very important in the study of the spread of behavior through complex networks. In order to theorize about this spread of behavior considering the network structure, let us consider a society with *n* individuals (nodes), within which interaction is described by a Poisson random network (Bollobás, 2001; Xu et al., 2014) with link probability p. This model can be seen as an adaptation of the unpublished Reed and Frost model (Bailey, 1975; Jackson, 2008). Let us assume that only one individual in the society has a behavior. According to the considerations used earlier in discussing the input–output system of an individual, we can determine this to be a self-induced behavior. This individual, who has an initial behavior, will interact with other people in the network (i.e., with his/her neighborhoods, in network terminology). Let us hypothesize that some other people are Immune to be affected by others' behavior, which is not unrealistic in real-world terms. In particular, let the probability that any individual is immune to be affected by others' behavior be represented by α. This probability can also be seen as the portion of the population that is naturally immune to be affected by others' behavior. Here, the spread of behavior can be modeled by generating a random Poisson network, using *n* nodes with p as the link probability; then, we delete uniformly, in a random way, αn nodes and eventually identify the component in which the first individual with the behavior can be located in such a sub-network (Jackson, 2008). Then, for this network, we analyze (1−α) *n* nodes and determine the size of the component by choosing a random node. Since the threshold for which a giant component emerges occurs when *p* (1−α) *n* = 1, we know that, when *p* (1−α) *n* < 1, we can determine that a small percentage of individuals will have that behavior. On the other hand, when *p* (1−α) *n* > 1, we expect that the behavior will spread to a portion of the susceptible individuals. In particular, f for *n* → ∞ (large n), when an individual lying in a “giant component” of the susceptible population is elicited with a stimulus producing a behavior, then the expected size of the diffusion as a rate of the nodes that are susceptible to stress is approximated by the r by solving *r* = 1−*e*^−*r*(1−α)*np*^. Moreover, if $p>\frac{log\left(\left(1-\alpha \right)n\right)}{\left(1-\alpha \right)n}$, then for *p* → 1, when *n* → ∞, the network of individuals susceptible to that behavior will be connected, and we will be sure that all such individuals actually will be with that behavior. Since r is difficult to determine, we can solve $\left(1-\alpha \right)np=\frac{log\left(1-r\right)}{r}$. This model can also be seen as a “percolation” through a network, after the removal of some nodes (Stauffer and Aharony, 1994; Grimmett, 1999). It is important to highlight that, until now, we have not considered that the transmission of a behavior is probabilistic, and, thus, the contagion cannot take place with certainty, in agreement with the results of our previous model. Thus, we now need to take this consideration into account. Now, we will generalize beyond the Poisson degree distributions. Let ${\langle d^{2}\rangle }_{\alpha }=2{\langle d\rangle }_{\alpha }$ be the threshold of percolation for an emerging giant component in the “configuration model” (Bender and Canfield, 1978; Wormald, 1984; Bollobás, 2001; Newman, 2001; Newman et al., 2001). Thus, ${\langle d^{2}\rangle }_{\alpha }=2{\langle d\rangle }_{\alpha }$ represents the point at which an individual with the behavior has a probability to influence an infinite number of other individuals f for *n* → ∞.

### Networked agent-based modeling

Modeling using agent-based objects^1^ proceeds from the bottom up in the understanding of complex systems. Practically, we create a computer program containing program parts representing artificial agents. By shaping these agents in an environment and endowing them with some rules, we let them interact each other over time in the so-called agent-based simulation, thus building an artificial laboratory in which to experiment with social and economic phenomena or whatever else we are interested to observe.

This new approach, unlike most mathematical and statistical models, allows us, for example, to build heterogeneous agents, to work in situations “far from equilibrium,” and to consider in the model the consequences of interactions among agents.

Agent-based models are becoming very popular in economic and social sciences; for example, in a review of a recent book on CAS by John H. Miller and Scott E. Page, the Nobel Prize in economics winner Kenneth J. Arrow said: “the use of computational, especially agent-based, models has already shown its value in illuminating the study of economic and other social processes” (Miller and Page, 2007). As highlighted by Gilbert (2008), a reason for this spread in the social sciences is that an agent-based simulation allows us to build models in which individual entities and their interactions are directly represented. This bottom-up approach is useful to understand the emergence of complex systems creating the interacting elements at a low level and looking for this emergence running simulations over time.

In an agent-based social simulation, we represent a sort of “social reality,” and we are not interested in inserting all features of a “real system” in our model, but we are interested in the few features, elements and interactions that allow us to observe the emergent phenomenon that we are trying to understand. Thus, we build our models practically from the bottom up.

According to this construction, we can think of agent-based simulations as experiments on complex systems, but some considerations are necessary. For example, many complex systems are experimented with agent-based, since these are such that it is not possible to do differently; for example, it is not possible to generate a real disaster to see its effect. Thus, an agent-based simulation is well-suited to the representation of such phenomena, since give us a practically instruments to manage the scenarios of behaviors (Table 4).

**Table 4:** **Modeling potential**.

**Traditional Tools**	**Agent-based Tools**
Precise	Flexible
Little process	Process-oriented
Timeless	Timely
Optimizing	Adaptive
Static	Dynamic
1, 2, … , or ∞	1, 2, … , N agents
Vacuous	Spaced/networked
Homogeneous	Heterogeneous

Adapted from Miller and Page (2007).

Agent-based simulations give us the possibility to change initial conditions, input, behaviors, interactions, structures, environments, and other “parameters” in order to have a large series of scenarios and not a single unique solution. This last consideration is both a significant limitation and one of the greatest resources of agent-based models.

Since agent-based is often modeled through code to run a simulation, we need to consider coding problems and errors in our model. Such errors are unavoidable, but thanks to the flexibility of agent-based modeling, the applications are wide and growing in the social sciences and many other fields. In fact agent-based modeling is conducted by many different kind of scientists, such as social scientist, engineers, philosophers, economists, psychologists, mathematicians, biologists, physicists, computer scientist, and sociologist. This is not surprising from a complex systems perspective; while science is certainly disciplinary, we have to remember that the “reality” is a unique one.

An agent-based model consist of many agents interacting in an environment. These agents can represent individual people, firms, nations, and other aggregates. These agents exchange information, rules, behaviors, and so on. An agent-based model differs from other computational models most distinctly in the possibility it offers of modeling an interaction between agents.

**Figure 9:**
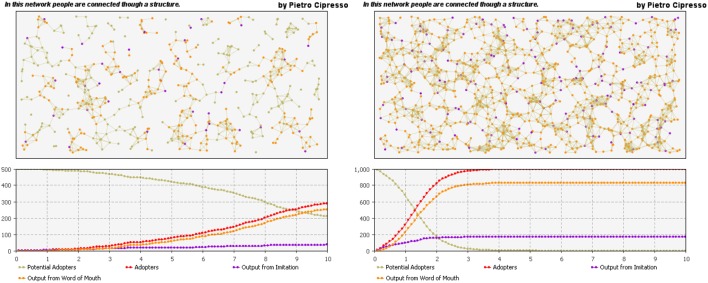
**Networked agent-based model**. The influence of network structure on behavior dynamics is huge, as can be seen by comparing the left-hand network with the more connected right-hand network.

The agents can also be represented and connected by the means of complex networks. The previous example of the behavior dynamics using SD can be also modeled by means of agent-based platforms, including the network structure through which the agents in the model are connected, as shown in Figure 9.

### Networked agent-based models formally explained

In an agent-based model, we define interaction among agents. Each agent has a “self-state” variable that provides information that each agent has or does not have. Moreover, the agent has a list of neighbors representing the list of agents to whom he is linked through networks.

Each agent can be endowed with a set of behaviors, depending on the phenomena that we want to study, and must decide whether to diffuse these behaviors to their neighbors and with what strength. This decision can be coded into the variable *behavioral_degree*, which is the result of the decision to diffuse a behavior with the degree (normalized to 0–1) assigned on the basis of the strength assigned. If *behavioral_degree* is positive, then the agent will try to spread the behavior to their neighbors; otherwise, it will not.

Let consider to be in a Barabasi-Albert network (i.e., a scale-free network) built iteratively in this way: from a small number of nodes (one), we add, at every time step, a new node with one edge that links it to an already present node i. This attachment is made preferentially with a probability of $\Pi \left(k_{i}\right)=\frac{k_{i}}{\underset{j}{\sum }k_{i}}$with *k*_*i*_ the degree of the node i. This construction provides a distribution of the degrees independent from the number of links a new node can have with the present nodes *P*(*k*) ≈ *k*^−α^, 2 ≤ α ≤ 3. It is possible to notice that this model is equivalent to the Susceptible-Infected model in a network. In a homogeneous network (with the same number 〈k〉 of vertices for all nodes), let us denote by *I* (*t*) the total number of nodes with the information; in that way, the proportion of nodes with the information *i* (*t*) is *I* (*t*)/*N* where *N* is the total number of nodes. The evolution equation of *i* (*t*) is then given, with λ being the diffusion probability of the information and (1−*i* (*t*)) being the proportion of nodes susceptible to receive the information. $\frac{di(t)}{dt}=\lambda i(t)\rangle k\langle (1-i(t)$ However, in a heterogeneous network, like ours, the average degree 〈k〉 is not relevant, so it is more convenient to compute *i*_*k*_(*t*) as the density of nodes having the information and the degree *k*: *i*_*k*_(*t*) = *I*_*k*_(*t*)∕*N*_*k*_ with *N*_*k*_ being the number of nodes with degree *k* and *I*_*k*_(*t*) being the number of nodes with degree *k* and with the information. In this case, the equation of density is as follows: $\frac{di_{k}\left(t\right)}{dt}=\lambda \theta_{k}\left(t\right)k\left(1-i_{k}\left(t\right)\right)$ θ_*k*_(*t*) (Boguná et al., 2003) (rather than *i* (*t*)) is the density of nodes with the information and also with neighbor of degree *k*, and (1−*i*_*k*_(*t*)) [rather than (1−*i* (*t*))] is the rate of nodes susceptible to receive the information and having a degree *k*. Barthélemy et al. (Barthélemy et al., 2004; Barthélemy, 2011) give a solution of the equation:

$$\begin{array}{l} i_{k}\left(t\right)=i_{0}\left[1+\frac{k\langle k\rangle }{\langle k^{2}\rangle -\langle k\rangle }\left(e^{t∕\tau }-1\right)\right] \end{array}$$

where $\tau =\frac{\langle k^{2}\rangle }{\lambda \left(\langle k^{2}\rangle \;-\;\langle k\rangle \right)}$ and *i*_0_ is the initial condition.

## Experimental study

The purpose of this experiment was to estimate the propensity to adopt and to imitate a behavior in a specific context. We used a sample of university students to analyze their behavior during examination, a very stressful situation for this sample. Recalling the previously mentioned definition of behavior from Gomez-Marin et al. (2014), we decided to focus on psychophysiological parameters to monitor behavioral change consequent to a stressor. The general aim of the study is to use experimental procedures to estimate the parameters that constitute the input of the computational model (SD model), in particular “Imitation Effectiveness” and “Adoption Fraction.” Once estimated, the computation model is able to estimate the dynamics of stress behavior in the student population. Ultimately, we are interested in looking at the gender differences between the two groups, in order to analyze the behavioral difference of male and female students in this situation (University examination).

**Figure 10:**
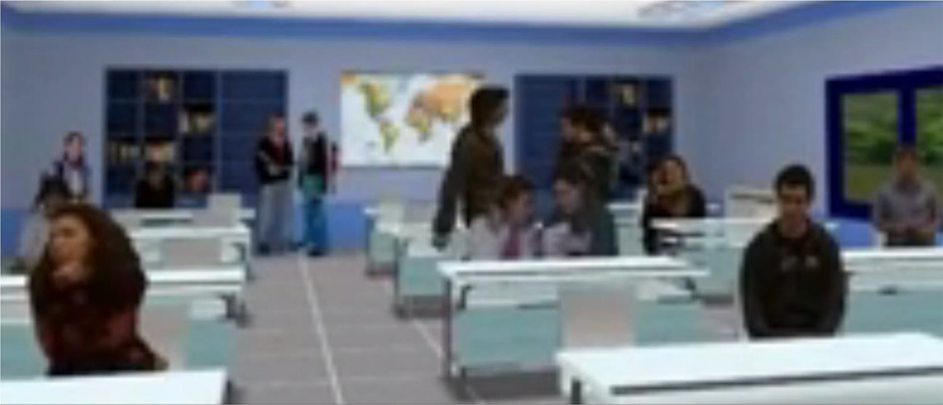
**A virtual classroom with real students (blurred for privacy reasons)**.

The “Adoption Fraction” is easy to experimentally estimate by using a cognitive task (such as a time pressure task) in order to induce stress. By doing this, we know the participant's exact propensity to stress in the presence of other stressed individuals. “Imitation Effectiveness” is more difficult to experimentally estimate, since we need to use an experience to provide a context within which the participant feels stressed because of other people's stress. For this purpose, we used different emotional induction techniques (namely, VR, Imagery Exposure, text, and video). Using an emotional narrative validated in a preliminary study (Raspelli et al., 2011), we created an audio, text, and video recording and a virtual environment based on that script (Figure 10). All four media in conjunction with the script have been found to be effective to elicit stress and behavioral responses (Pallavicini et al., 2013).

A sample of the script used in this study is as follows: “It's your last exam before graduating. The next session for this exam will not be held till next year. You know that if you fail, you will have to wait several months to graduate. You feel very nervous. In the exam room, you see four of your classmates. They are talking about the examination, and they seem to be very worried and troubled. They tell you that all of them failed the exam and that the professor is very strict. Your anxiety increases. You feel your breath becoming heavy. Your heart is beating faster and faster (…).”

### Participants

The study included a sample of 42 right-handed undergraduate students (26 females and 16 males, everyone with 14 years of education) from a university's introductory psychology class. Three subjects were excluded from experiments due to technical problems associated with the complexity of the instruments involved in this study.

### Materials and methods

The study involved the use of two questionnaires: the Post Media Questionnaire (PMQ) (Ray, 2007) and the Slater-Usoh-Steed Presence Questionnaire (SUS) (Slater et al., 1994). The study also involved many physiological measures: EMG-Z, Facial electromyography Zygomatic; EOG, Electrooculography; ECG/EKG, Electrocardiography; RSP, Thoracic (chest) Respiration; SC/GSR, Skin Conductance; EEG, 14 Electroencephalography channels (plus CMS/DRL references, P3/P4 locations). These EEG channels (based on the international 10–20 location system) are: AF3, F7, F3, FC5, T7, P7, O1, O2, P8, T8, FC6, F4, F8, AF4.

### Experimental design

Before beginning the experiment, A 3 min baseline measurement was collected. During this measurement, physiological parameters were recorded. Because participants in the VR condition used their right hand to move around in the VR environment using a joystick, the baseline for the VR condition had be recorded during a virtual navigation task through an empty, neutral space in order to control for hand movement. The sequence of the experimental conditions was randomized prior to beginning the experiment. The conditions are as follows:

- *Condition 1* : VR. In the VR condition, subjects were asked to wear a HMD in order to have a 3-D view of the virtual environment. Subjects were also given a joystick to allow them to explore and interact with the scene. The virtual environment represented an academic oral examination in an auditorium. The virtual scene is included in NeuroVr 2.0.
- *Condition 2*: Imagery Exposure (AUDIO). Following an audio narrative, subjects were asked to close their eyes and imagine themselves as vividly as possible during an academic oral examination. Script development procedures were based on methods developed by (Lang et al., 2000) and adopted in a previous study of Raspelli et al. (2011).
- *Condition 3*: Text (TEXT). Based on the script used in the Imagery Exposure condition, a 2 min script was developed in written form. Participants were asked to read a text in which an academic oral examination was described in detail (Gerrig, 1993).
- *Condition 4*: Video (VIDEO). In the VIDEO condition, subjects were exposed to a video based on the previous script about an academic oral examination.
- *Condition 5*: A Cognitive Task (a time pressure task) was assigned to participants in order to induce stress.

A 5 min break was included between all sessions.

The collected data were used to estimate adoption fraction and imitation effectiveness. In particular, adoption fraction was estimated in condition 5, and imitation effectiveness was estimated in conditions 1–4.

### Procedures

Subjects were informed that they were participating in a study regarding the effects of mental imagery on physiological arousal. They were asked not to smoke or consume caffeine on the day of the experiment to avoid any effects of these substances on the central autonomic nervous system. Exclusion criteria were related to the states of their cardiac, mental and psychological health. Subjects who met the experimental criteria were contacted via email and/or telephone to schedule a meeting at the lab. Each subject was asked to provide written consent for inclusion in the investigation. Researcher explained the broad function of electrodes and the subjects were prepared in order to collect the psychophysiological indexes. At the end of the experimental session, the experimenter helped subjects to remove all electrodes and patches, while explaining the scientific rationale for the use of the stimuli and the aims of the experiment.

### Algorithmic procedures

Every recording was marked through a synchronization algorithm developed (by using Matlab) for the alignment of the stimulus with all the psychophysiological signals (Cipresso et al., 2010). Furthermore, this algorithm allow us to synchronize EEG with all other psycho-physiological signals; since these two series of signals was recorded using different devices, it was necessary to align them. To improve the precision of the algorithm, the subjects were asked to blink their eyes rapidly five time before each stimulus; using electrodes near the eyes (Electrooculogram or EOG) connected to all other biosensors, as well as EEG with eye blink detection, this operation guaranteed precision to 1/100 of s, thus allowing for better analysis and success of experiment.

Once extracted, all biosignals were managed in Matlab and branched into six categories: *Baseline, Audio, Text, Video, VR*, and *Cognitive task*. Each category contains all EEG and psychophysiological signals of that session and can be prepared for signal processing procedure in order to extract a series of indexes for the statistical analysis.

Respiration signal can be elaborated to compute the Respiration Depth (RSP Depth), the point of maximum inspiration minus the point of maximum expiration, as determined by respiratory tracing. Smaller values indicate more shallow respiration and higher activation (Kunzmann et al., 2005; Mauri et al., 2010). It is also possible to calculate Respiration rate (also measured in breaths per minute) using peak-to-peak computing.

Cardiovascular activity was monitored to evaluate both voluntary and autonomic effects of respiration on heart rate in both physical and virtual environment interactions, analyzing both R-R interval (from the ECG) and respiration (from the chest strip sensor) and their interaction. Furthermore, standard HRV spectral method indexes and the like can be used to evaluate the autonomic nervous system response (Camm et al., 1996; Magagnin et al., 2010; Mauri et al., 2011). Cardiovascular and respiratory activity interaction can also be taken into account through the Respiratory Sinus Arrhythmia (RSA) index (Camm et al., 1996; Magagnin et al., 2010). Temporal domain measures of heart rate variability are generally calculated in term of AVNN, SDNN, RMSSD, and NN50 indexes (Table 5). To simplify, NN intervals can be seen as a beat-to-beat representation of heart rate; according to Camm et al. (1996), “In a continuous ECG record, each QRS complex is detected, and the so-called normal-to-normal (NN) intervals (that is, all intervals between adjacent QRS complexes resulting from sinus node depolarization) or the instantaneous heart rate is determined.”

**Table 5:** **Indexes used for the estimation of ImitationEffectiveness and AdoptionFraction**.

**Sensor**	**Index code**	**Index explanation**
EMG	EMG-Z	Facial Electromyography zygomatic index
EMG	EMG-CS	Facial Electromyography corrugator index
GSR	SC_Mean	Mean value of Skin Conductance extracted from GSR
RSP	RSP_Amp	Respiration amplitude mean
RSP	RSP_Period	Respiration period mean
ECG + RSP	RSA	Respiratory sinus arrhythmia index
ECG	LFbyHF	LF/HF Ratio of low to high frequency power (Sympathovagal balance)
ECG	HF	HF Total spectral power of all NN intervals between 0.15 and 0.4 Hz
ECG	LF	LF Total spectral power of all NN intervals between 0.04 and 0.15 Hz
ECG	TOTPWR	Total spectral power of all NN intervals up to 0.04 Hz
ECG	pNN50	Percentage of differences between adjacent NN intervals that are greater than 50 ms
ECG	rMSSD	Square root of the mean of the squares of differences between adjacent NN intervals
ECG	SDNN	Standard deviation of all NN intervals
ECG	AVNN	Average of all NN intervals
EEG	Alpha	Spectral power of EEG in the range 8–12 Hz
EEG	Beta	Spectral power of EEG in the range 13–30 Hz
EEG	EEG_Asym	EEG activation asymmetry measured as prevalence of alpha waves in the right hemisphere
Self-report	SUS_Post-Bas	Slater-Usoh-Steed Presence Questionnaire (SUS) [POST STIMULUS-BASELINE] (to check presence existence)
Self-report	PMQ_Anxiety_Post-Pre	Post Media Questionnaire (PMQ-ANXIETY SCALE) [POST STIMULUS-PRE STIMULUS]
Self-report	PMQ_Relax _Post-Pre	Post Media Questionnaire (PMQ-RELAX SCALE) [POST STIMULUS-PRE STIMULUS]

Spectral analysis was performed by means of Fourier spectral methods with custom software. The rhythms were classified as very low frequency (VLF, < 0.04 Hz), low-frequency (LF, from 0.04 to 0.15 Hz) and high frequency (HF, from 0.15 to 0.5 Hz) oscillations. The power will be expressed in absolute (LF_RR_, LF_resp_ and HF_RR_, HF_resp_) and in normalized units. For example, in RR series: LFnu and HFnu as 100*LF_RR_/(σ^2RR^-VLF_RR_) and 100*HF_RR_/(σ^2RR^-VLF_RR_), where σ^2RR^ represents the RR variance and VLF_RR_ represents the VLF power expressed in absolute units.

Skin conductance (SC) mean can be extracted from a GSR biosensor. It is critical to remove possible movement artifacts before computing the index (since, on the hand, it can be affected by consistent involuntary grasping). SC is an interesting measure, since the sweat glands are regulated by the sympathetic nervous system without a direct “contamination” of the parasympathetic nervous system (which exists, for example, for HR). Thus, SC is an excellent candidate to measure pure physiological arousal (Fowles et al., 1981).

Central nervous system indexes for stress, evaluated through EEG, using the global beta (13–30 Hz), as indicated in the literature, have a long tradition and have been used in many study (Nikulin and Brismar, 2004; Bagić et al., 2011a,b). However, other interesting indexes have also been used in recent literature. In particular, the frontal EEG activation asymmetry has been used to show that greater left frontal activity seems to be more highly related to positive mood, whereas greater right frontal activity seems to be more involved in negative moods, such as stress. Though more studies are required, there are indications that greater right hemisphere activity is caused by increased levels of stress and decreased level of immune functioning. Furthermore, according to other interesting studies (Gotlib et al., 1998; Urry et al., 1999; Debener et al., 2000; Diego et al., 2001; Niemiec and Lithgow, 2005; Tops et al., 2005; Nusslock et al., 2007, 2008; De Raedt et al., 2008; Mathersul et al., 2008; Gordon et al., 2010), there is evidence of higher cortisol levels in individuals with greater right frontal activity, and, according to many authors, more cortisol is released from the right hemisphere than from the left. Following the recent literature, we used Alpha waves (8–12 Hz) that seem to be the best suited to studying the frontal EEG activation asymmetry.

The raw electromyography (EMG raw) is a collection of positive and negative electrical signals; their frequency and amplitude give us information on the contraction or rest state of the muscle. Amplitude is measured in μV (micro-Volts). As the subject contracts the muscle, the number and amplitude of the lines increase; as the muscle relaxes, the same decrease (Goodmurphy and Ovalle, 1999; Veldhuizen and Gaillard, 2001; Larsen et al., 2003). Previous study considered the Root Mean Square (RMS) for rectifying the raw signal and converting it to an amplitude envelope (Blumenthal et al., 2005; Mauri et al., 2010). In certain cases, we can also focus on frequency, related to muscle fatigue (Veldhuizen and Gaillard, 2001). A number of measures can be extracted from this signals depending on the muscle corresponding to the electrodes' locations. For the model, there are three facial locations that give relevant information about emotional valence. In particular, RMS of EMG signal recorded in correspondence of facial zygomatic major muscle (following EMG Zygomatic), increases when positive emotions arises (Larsen et al., 2003; Blumenthal et al., 2005). On the other hand, the RMS of EMG signal recorded in correspondence with facial corrugator supercilii muscle (following EMG Corrugator), increases when negative emotions arises (Larsen et al., 2003; Blumenthal et al., 2005).

### Results

Results of the two parameters (ImitationEffectiveness and AdoptionFraction) depend on the stress threshold that we use (Table 6). A higher threshold means that the parameters will be affected when the stress level is very high. On the other hand, the use of a lower threshold also affect the parameters in the case of medium or low stress. The two parameters have values theoretically ranging from 0 (lowest stress threshold) to 1 (highest stress threshold).

**Table 6:** **Code for computation of ImitationEffectiveness and AdoptionFraction variables**.

**INPUT** (“Stress Threshold,” ST, %); **IF** {[Δ(EMG-Z) < ST] OR [Δ(EMG-CS) > ST]} AND [Δ(SC_Mean) > ST]} OR { [Δ(RSA) < ST] OR [Δ(LFbyHF) > ST] OR [Δ(HF) < ST] OR [Δ(LF) > ST] OR [Δ(TOTPWR) > ST] OR [Δ(pNN50) < ST] OR [Δ(rMSSD) < ST] OR [Δ(SDNN) < ST] OR [Δ(AVNN) < ST] } OR { n-Tuple (couple, triple, …) combination of the following rules: Δ(RSA) < ST] OR [Δ(LFbyHF) > ST] OR [Δ(HF) < ST] OR [Δ(LF) > ST] OR [Δ(TOTPWR) > ST] OR [Δ(pNN50) < ST] OR [Δ(rMSSD) < ST] OR [Δ(SDNN) < ST] OR [Δ(AVNN) < ST] OR [Δ(Alpha) < ST] OR [Δ(Beta) > ST] OR [Δ(EEG_Asym) < ST] } OR [Δ(SUS_Post-Bas) > 0] OR [Δ(PMQ_ANXIETY_Post-Pre) > ST] OR [Δ(PMQ_RELAX_Post-Pre) < ST]; **THEN** Δ(ImitationEffectiveness) > 0; *considering indexes in conditions 1–4 Δ(AdoptionFraction) > 0; *considering indexes in condition 5 **OUTPUT** (ImitationEffectiveness AdoptionFraction);

In general, the choice of stress threshold is a key element, since the simulation outputs can be considered only for that specific stress level. Thus, when we speak about population diffusion of stress in a simulation, we mean only for that level of stress that we considered in the threshold (e.g., very stressful events; 95th percentile of stress level). Indeed, this key aspect can also be considered as a potential instrument to assess how the different stress levels considered can affect the diffusion of the stress among the population, as well as to consider differences among groups.

In the present study, for example, we can consider two groups by gender (male and female) to analyze whether and how stress diffuses differently between male and female subjects.

**Figure 11:**
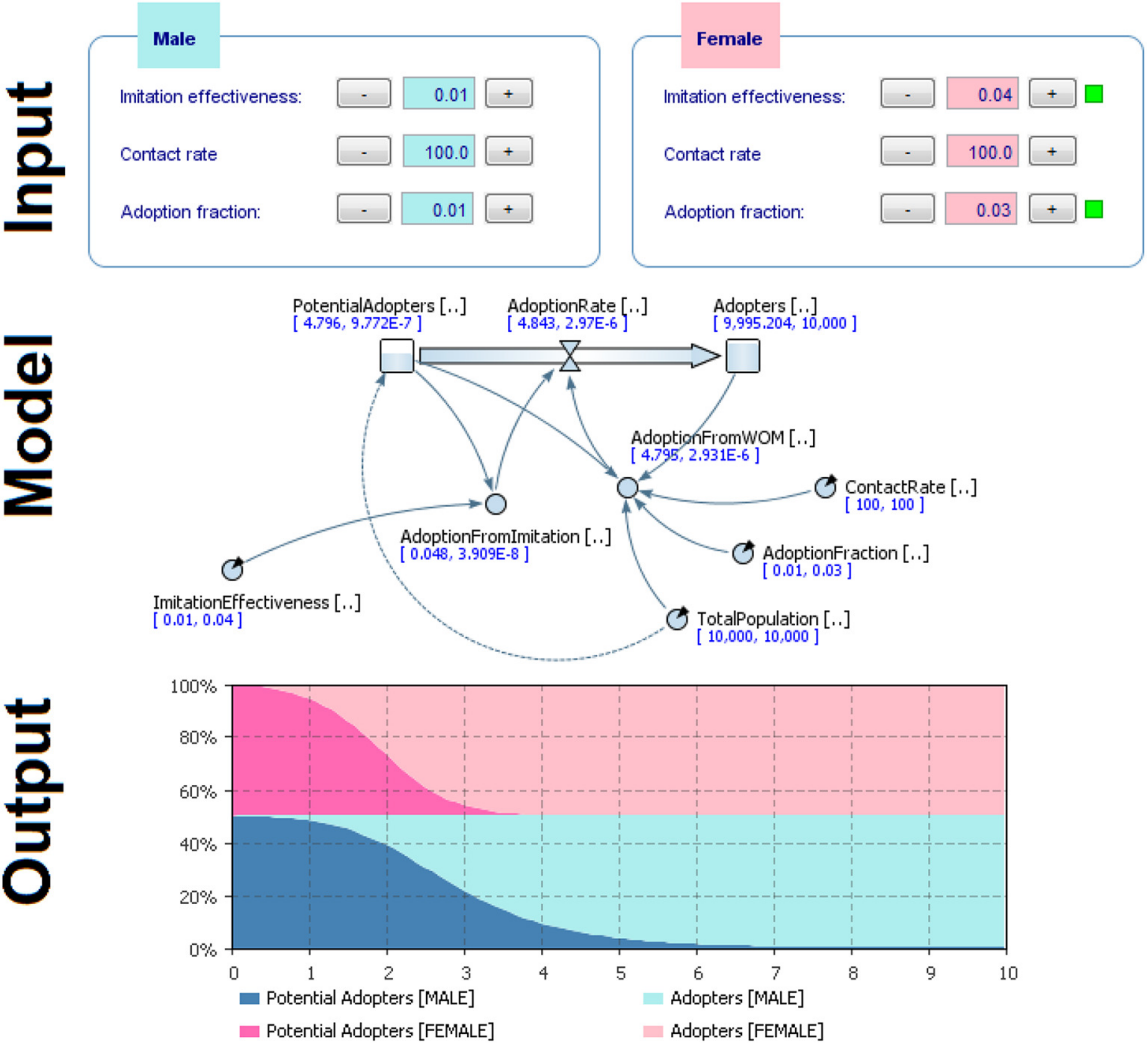
**Results of comparison of the two groups (male vs. female) with different initial conditions to model behavior dynamics**.

Considering the high level of stress threshold defined previous, we obtained the following parameters (Figure 11, Input), which applied to the simulation model (Figure 11, Model), gave the following output as a result (Figure 11, Output).

**Figure 12:**
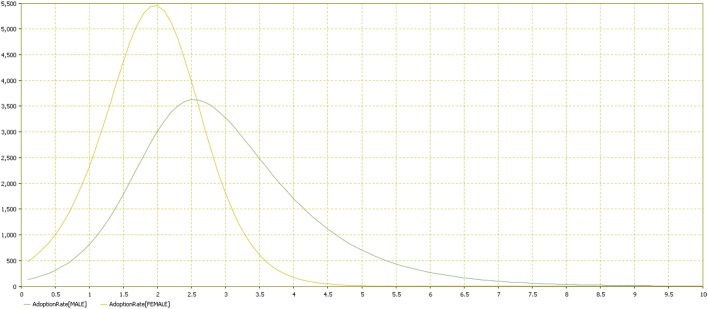
**Adoption rate distribution by gender**. Comparison of the two groups (male vs. female). Adoption rate is reported in the vertical axis by time in the horizontal one.

**Figure 13:**
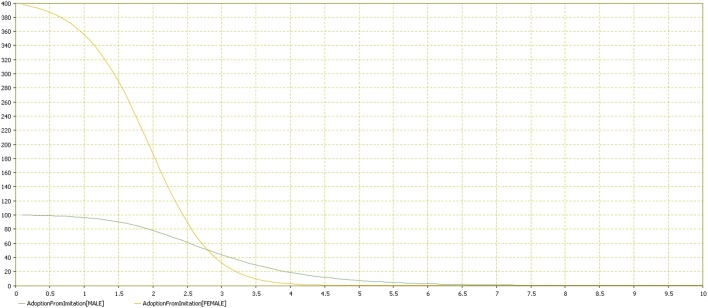
**Adoption from imitation distribution by gender**. Comparison of the two groups (male vs. female). Adoption from imitation is reported in the vertical axis by time in the horizontal one.

**Figure 14:**
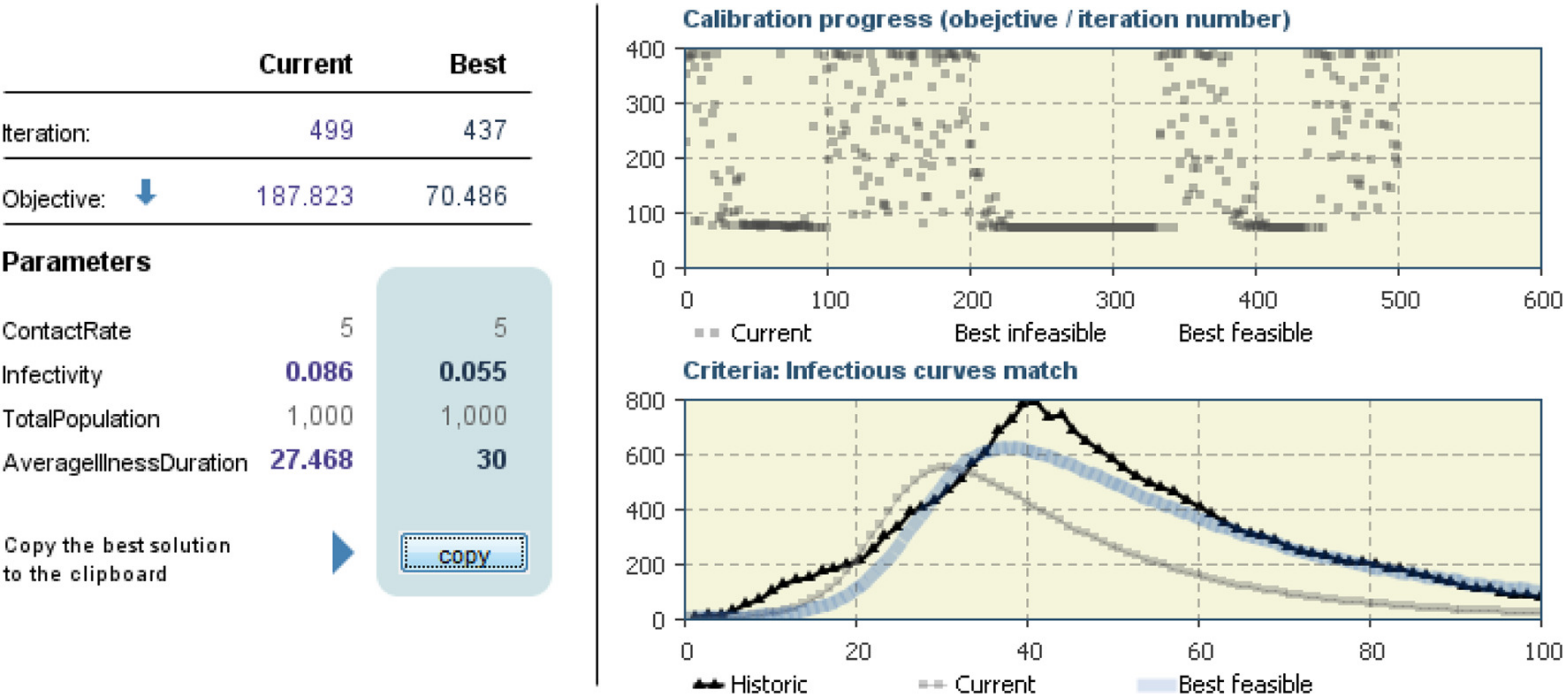
**Calibration of SIR model with extrapolation of parameters to be used in the SIR model**.

**Figure 15:**
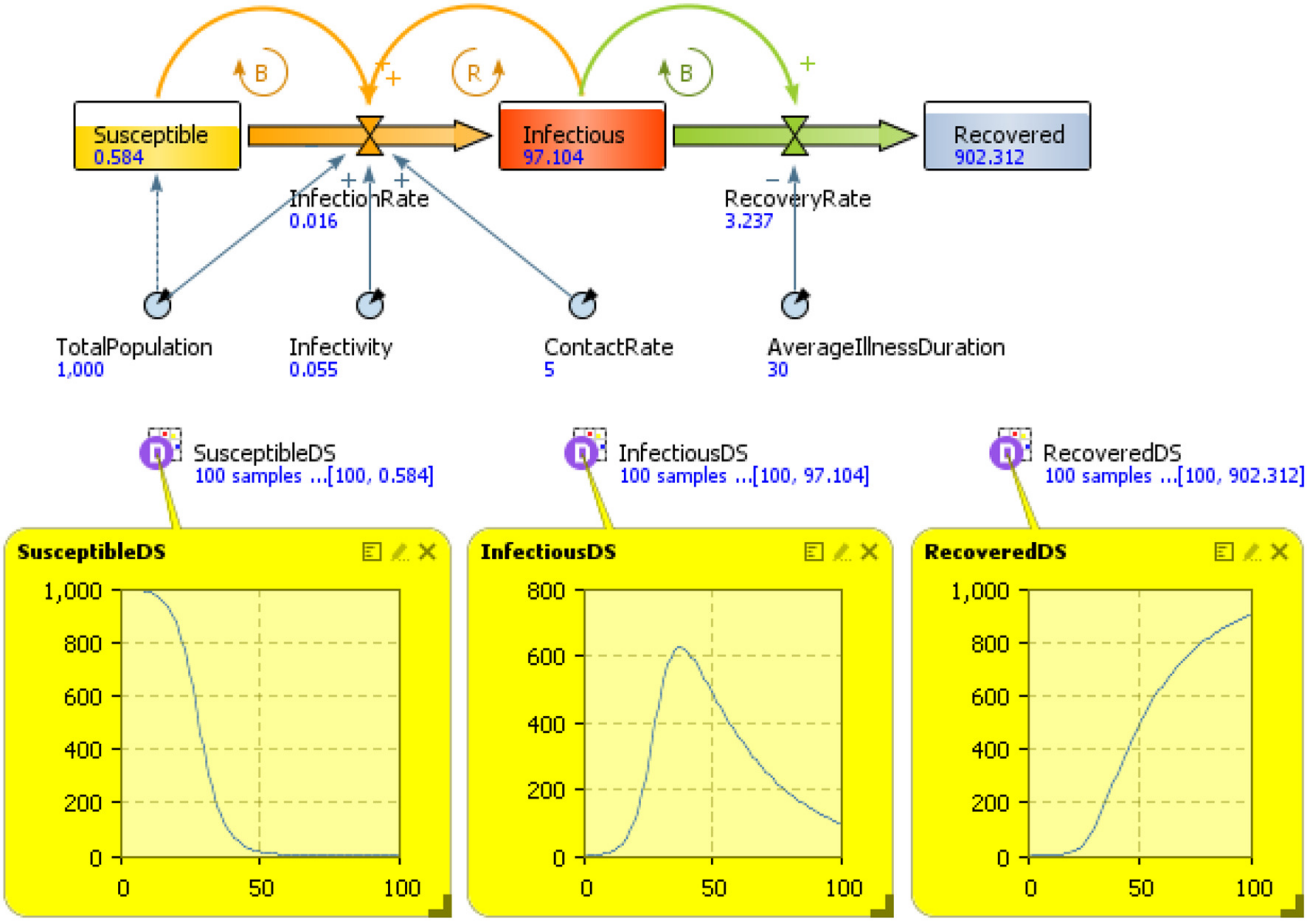
**SIR Model with input parameter arose from calibration**.

Results showed that females adopted the behavior more quickly (Figures 11–13). Practically, accordingly to this data, during an examination, in a classroom with all females there will be a stress behavior diffusion in about the half of the time than in a class with all males. We do not have epidemiological historical data for the SIR model described previously; however, simulating a time series, we obtained the calibration to be used as an input parameter for the model (Figures 14, 15).

## Promoting behavioral change: an integrated approach

**Figure 16:**
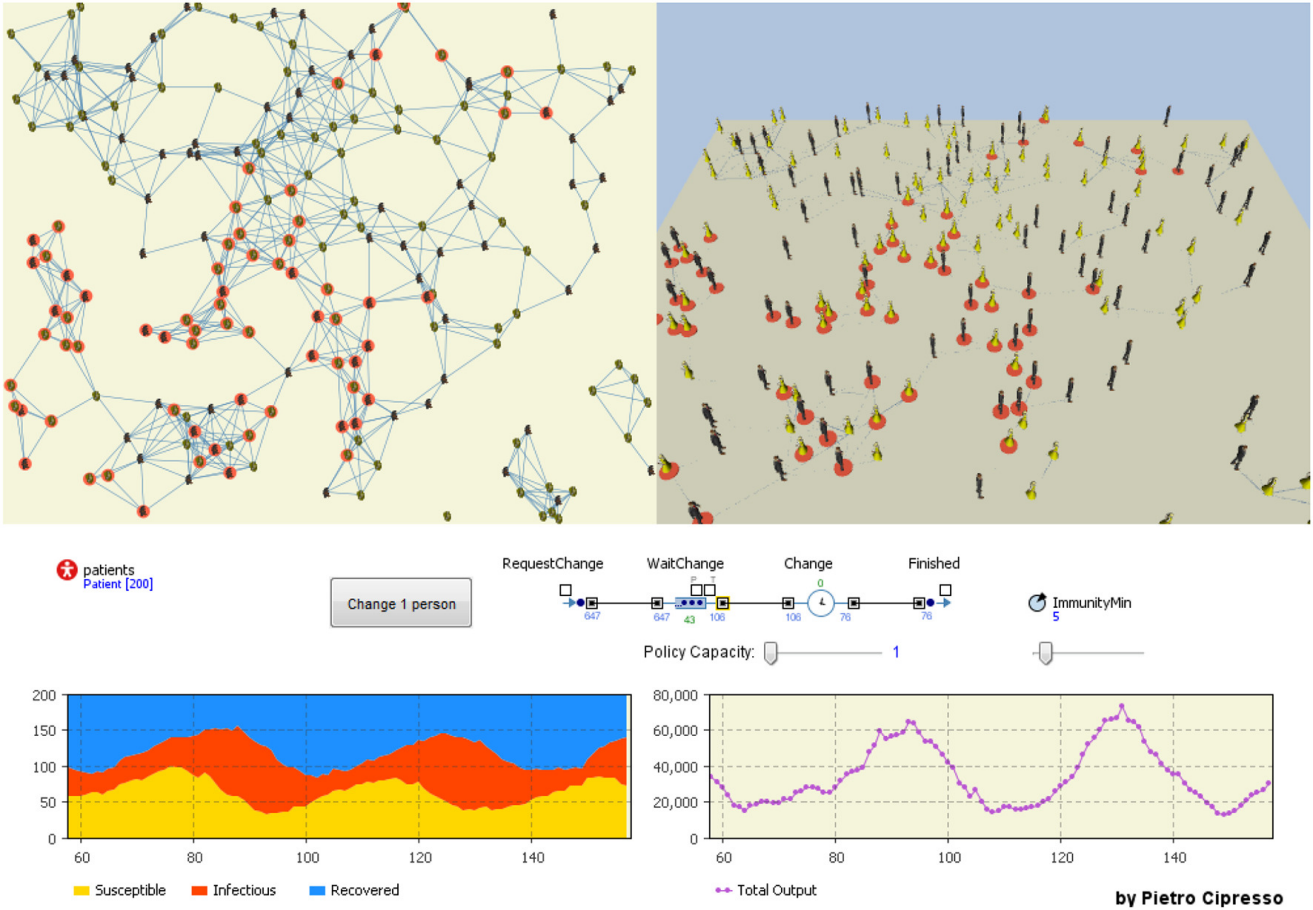
**The integrated model considering networked agents, system dynamics models and discrete event models to govern policy capacity (effectiveness)**.

The following model (Figure 16) is based on SIR, as previously defined, where people are modeled as artificial agents and their behavior output is measured as SD. When these outputs become higher than a given threshold, a behavior change is requested by the artificial agents (which can be seen as a request for help to change behavior in psychological terms). The way in which behavior is changed is modeled through a top-level Discrete Event model (which can be seen as the public policy). The model has multiple objectives, including that of defining the behavior dynamics following the policy that promote some behavioral change.

The idea of creating an integrated model has a practical application when we consider computational models as tools for management. For example, let us consider a company using simulation to forecast scenarios that help managers in making decision. In this model, all variable can be set to consider a specific behavior dynamic (e.g., absenteeism). Let us suppose the company wants to change employees' hierarchical positions, representing a *de facto* change in the network structure of the company. This model would be able to estimate an increase or decrease in absenteeism following managers' decisions by creating a wide range of possible scenarios among which to choose and make decisions.

Using VR can be also a huge boost for computational modeling. NeuroVirtual 3D can be used to interact in real time with behavior simulation. Such a method represents a brand new way to build more effective predictions of behavior dynamics. Integrating user data, behavioral data (with VR), relational data (with social network analysis) and physiological data (with biosensors during VR behavior), it is possible to connect real, artificial and virtual worlds for a realistic modeling of behavior dynamics. Moreover, the results of the model can contribute back to shape the virtual world, creating a closed loop between real and simulated behavior.

## Conclusions

This article introduced a brand new method for modeling behavior. While there is a long tradition of modeling behavior by using mathematical tools, their integration with computational methods has been always done by disentangling models from real behavior. Computational psychometrics, by using measures of real behavior during realistic situations within VR, provides a more effective interconnection between real world and computational models, even if with the limitation of being a new method to be tested with specific behavior dynamics. Being VR very validated in experimental and clinical settings, there is the safety that exposure works better than mental imagery, but of course this depends on the situations elicited: it is licit to think to some for which VR doesn't works (Pallavicini et al., 2013).

The spread of low-cost technologies now makes it possible to create computational simulations within a lab by using a commercial computer and free or low-cost software. Moreover, the increased capability to easily record large amount of behavioral data (Gomez-Marin et al., 2014) at a low cost, thanks to new technologies and ubiquitous computing (with devices such as smartphones), constitutes a way to further link real and simulated behavior.

Applications range from economics to medicine, decision-making, management, psychology, engineering, and many others. To model behavior dynamics provides the ability to build realistic scenarios, giving the power to policy makers to check the consequence of their decisions. Moreover, the closed loop presented here allows for a real-time verification and modification of the behavior dynamics models, which also contributes to changes in real behaviors toward a systemic homeostasis and an effective way to manage decisional processes in a range of behavioral actions.

### Conflict of interest statement

The author declares that the research was conducted in the absence of any commercial or financial relationships that could be construed as a potential conflict of interest.

## Footnotes

^1^According to Miller and Page (2007), this could be a better name with which to identify agent-based models; furthermore, it is useful to distinguish bottom-up modeling (e.g., artificial adaptive agents) from bottom-up simulation (e.g., artificial life).

## Supplementary material

Supplementary Material **All the models in this manuscript have been uploaded as a Data Sheet (.zip file)**. To be executed, the models require an updated version of Java. I created a .bat file, which is the one that should be executed. The reviewers can run the models as simulations without the need to connect with NeuroVirtual 3D simulations or other software. It is possible to change thresholds to see how the chosen ones change the simulations.
