# Supplementary material for: Modeling behavior dynamics using computational psychometrics within virtual worlds
Source: Front Psychol. 2015 Nov 6;6:1725. doi: 10.3389/fpsyg.2015.01725 (PMC4635205; doi:10.3389/fpsyg.2015.01725)
Supplement: Supplementary Material — All the models in this manuscript have been uploaded as a Data Sheet (.zip file). To be executed, the models require an updated version of Java. I created a .bat file, which is the one that should be executed. The reviewers can run the models as simulations without the need to connect with NeuroVirtual 3D simulations or other software. It is possible to change thresholds to see how the chosen ones change the simulations. [file DataSheet1.ZIP › Behavior Dynamics (Bass Model using SD)/Spread Model (SD).html]

xml version="1.0" encoding="UTF-8"?


Spread Model (SD) - Simulation


If the applet doesn't start successfully, please run 'Spread Model (SD).bat' for Windows, or 'Spread Model (SD).sh' for Mac or Linux, which are located in the same folder.

<applet
code="bass\_diffusion/Simulation$Applet.class"
archive="com.xj.anylogic.engine.jar,model.jar"
width="902"
height="658">
</applet>
