# Supplementary material for: Modeling behavior dynamics using computational psychometrics within virtual worlds
Source: Front Psychol. 2015 Nov 6;6:1725. doi: 10.3389/fpsyg.2015.01725 (PMC4635205; doi:10.3389/fpsyg.2015.01725)
Supplement: Supplementary Material — All the models in this manuscript have been uploaded as a Data Sheet (.zip file). To be executed, the models require an updated version of Java. I created a .bat file, which is the one that should be executed. The reviewers can run the models as simulations without the need to connect with NeuroVirtual 3D simulations or other software. It is possible to change thresholds to see how the chosen ones change the simulations. [file DataSheet1.ZIP › Behavior Dynamics (Integrated Model)/Behavior Change (Integrated model).html]

xml version="1.0" encoding="UTF-8"?


Behavior Change (Integrated model) - Simulation


If the applet doesn't start successfully, please run the 'model.jnlp' file. Applets with 3D animation may fail to run in the latest versions of Java. In that case please consider placing your applet on a web server or using an older version of JRE.


<applet
code="org.jdesktop.applet.util.JNLPAppletLauncher"
archive="lib/applet-launcher/applet-launcher.jar,lib/jogl/jogl.jar,lib/gluegen/gluegen-rt.jar,com.xj.anylogic.engine.jar,com.xj.anylogic.engine.al3d.jar,lib/aviatrix3d-all\_2.0.0.jar,lib/j3d-org-elumens.jar,lib/j3d-org-geom-core.jar,lib/j3d-org-geom-hanim.jar,lib/j3d-org-geom-particle.jar,lib/j3d-org-geom-terrain.jar,lib/j3d-org-loader-3ds.jar,lib/j3d-org-loader-core.jar,lib/j3d-org-loader-dem.jar,lib/j3d-org-loader-stl.jar,lib/j3d-org-loader-vterrain.jar,lib/j3d-org-navigation.jar,lib/j3d-org-texture.jar,lib/j3d-org-util.jar,lib/javax.vecmath\_1.5.0.jar,lib/uri.jar,lib/vlc\_uri.jar,lib/xj3d-common\_2.0.0.jar,lib/xj3d-config\_2.0.0.jar,lib/xj3d-core\_2.0.0.jar,lib/xj3d-device\_2.0.0.jar,lib/xj3d-eai\_2.0.0.jar,lib/xj3d-ecmascript\_2.0.0.jar,lib/xj3d-external-sai-concrete\_2.0.0.jar,lib/xj3d-external-sai\_2.0.0.jar,lib/xj3d-images\_2.0.0.jar,lib/xj3d-java-sai-concrete\_2.0.0.jar,lib/xj3d-java-sai\_2.0.0.jar,lib/xj3d-jaxp\_2.0.0.jar,lib/xj3d-jsai\_2.0.0.jar,lib/xj3d-net\_2.0.0.jar,lib/xj3d-norender\_2.0.0.jar,lib/xj3d-ogl\_2.0.0.jar,lib/xj3d-parser\_2.0.0.jar,lib/xj3d-render\_2.0.0.jar,lib/xj3d-runtime\_2.0.0.jar,lib/xj3d-sai-concrete\_2.0.0.jar,lib/xj3d-sai\_2.0.0.jar,lib/xj3d-sav\_2.0.0.jar,lib/xj3d-script-base\_2.0.0.jar,lib/xj3d-xml-util\_2.0.0.jar,lib/xj3d-xml\_2.0.0.jar,model\_3d\_files.jar,model.jar,lib/ProcessModelingLibrary.jar"
width="1002"
height="758">
<param name="codebase\_lookup" value="false">
<param name="subapplet.classname" value="epidemic\_and\_clinic\_with\_accumulating\_concern.Simulation$Applet">
<param name="subapplet.displayname" value="Behavior Change (Integrated model) - Simulation">
<param name="noddraw.check" value="true">
<param name="progressbar" value="true">
<param name="jnlpNumExtensions" value="1">
<param name="jnlpExtension1" value="http://www.runthemodel.com/javanetcache/media/jogl/builds/archive/jsr-231-1.1.1/webstart/jogl.jnlp">
<param name="java\_arguments" value="-Dsun.java2d.noddraw=true">
<param name="jnlp\_href" value="model.jnlp">
</applet>
